# Mechanistic insights into the attenuation of intestinal inflammation and modulation of the gut microbiome by krill oil using in vitro and in vivo models

**DOI:** 10.1186/s40168-020-00843-8

**Published:** 2020-06-04

**Authors:** Fang Liu, Allen D. Smith, Gloria Solano-Aguilar, Thomas T. Y. Wang, Quynhchi Pham, Ethiopia Beshah, Qingjuan Tang, Joseph F. Urban, Changhu Xue, Robert W. Li

**Affiliations:** 1 College of Food Science and Engineering, Ocean University of China, Qingdao, China; 2 United States Department of Agriculture, Beltsville Human Nutrition Center, Diet, Genomics and Immunology Laboratory, Beltsville, MD USA; 3 United States Department of Agriculture, Agricultural Research Service, Animal Genomics and Improvement Laboratory, Beltsville, MD USA

**Keywords:** Krill oil, Colitis, Metabolome, Microbiome, Helminth, *Citrobacter rodentium*, Microbial signature, Balance

## Abstract

**Background:**

The anti-inflammatory property of ω-3 polyunsaturated fatty acids (PUFA) has been exploited in the management of inflammatory bowel disease (IBD) with promising results. However, it remains unclear if PUFA play a significant role in the resolution of inflammation and promotion of mucosal healing. Krill oil (KO) is a natural product rich in PUFA and the potent antioxidant, astaxanthin. In this study, we attempted to understand the mechanisms through which KO modulates the gut microbiome and metabolome using in vitro and in vivo colitis models and a multi-omics based approach.

**Results:**

KO significantly decreased LPS-induced IL1β and TNFα expression in human macrophages in vitro in a dose-dependent manner by regulating a broad spectrum of signaling pathways, including NF-κB and NOD-like receptor signaling, and displayed a synergistic effect with COX2 and IKK2 inhibitors in attenuating inflammatory pathways. Moreover, KO was involved in the resolution of inflammation by promoting M2 polarization and enhancing macrophage-mediated intracellular bacterial killing. Parasite-dependent intestinal mucosal damage and microbial dysbiosis induced by *Trichuris suis* infection in pigs were partially restored by feeding KO. KO supplementation reduced the abundance of *Rickettsiales* and several species of *Lactobacillus*, which were among the important features identified by random forests analysis contributing to classification accuracy for KO supplementation. Several microbial signatures with strong predictive power for the status of both infection and supplementation were identified. The inhibitory effect of KO on histidine metabolism was identified using untargeted metabolomics. KO supplementation reduced several key metabolites related to histamine metabolism by suppressing the expression of a gene encoding l-histidine decarboxylase in the colon mucosa and reducing histamine biosynthesis of microbial origin. Moreover, the pro-resolving properties of KO were validated using a *Citrobacter rodentium*-induced Th1-dependent colitis murine model. Further, microbial signatures with high prediction accuracy for colitis-related pathophysiological traits were identified in mice.

**Conclusion:**

The findings from this study provided a mechanistic basis for optimizing microbiome-inspired alternative therapeutics in the management of IBD. The microbial signatures identified, particularly those with strong predictive accuracy for colitis phenotypes, will facilitate the development of biomarkers associated with appropriate dietary intervention to manage intestinal inflammation.

Video abstract.

## Background

Inflammatory bowel disease (IBD) is a disease of global concern with a growing prevalence in children and young adults. Numerous intertwined biotic and abiotic factors, including host genetics, immunity, diet, gut microbiome, and environmental variables, play important roles in the pathogenesis of IBD [1]. In spite of the currently available therapeutic options for IBD, which include antibiotics, aminosalicylates, corticosteroids, immunosuppressants, and novel biologics that target TNFα, the medical needs still remain unmet. A range of adverse effects associated with these drugs have been documented [2, 3]. Novel biologics are designed to reduce these side effects. However, limited initial responsiveness and the high cost associated with biologics are a serious concern [1, 2]. Because of these issues, a growing number of IBD patients are turning to complementary and alternative strategies for help.

Recently, microbiome-inspired therapeutics, such as fecal microbiome transplantation, and natural products with potent anti-inflammatory properties, have been promoted as viable alternatives to conventional therapeutics [4]. Numerous nutritional and dietary strategies have been developed to aid the treatment of IBD [5]. For example, ω-3 polyunsaturated fatty acids (n-3 PUFA) have been extensively evaluated to prevent and treat IBD [4, 6]. Though their effects on clinical end points, such as the maintenance of remission, relapse rates, or disease activity indices of ulcerative colitis (UC) and/or Crohn’s disease (CD), are still debatable, the ability of n-3 PUFA to attenuate intestinal inflammation is seemingly incontestable and has been observed in several clinical trials (reviewed in [7]).

Krill oil (KO) is extracted from the Antarctic small crustacean species, *Euphausia superba* [8]. KO is rich in n-3 PUFA, such as eicosapentaenoic acid (EPA) and docosahexaenoic acid (DHA), which represent more than 31% of the total weight. Further, KO contains a potent antioxidant, astaxanthin (Supplementary Table S1). One of the major advantages of KO over traditional fish oil lies in the readily available delivery of PUFA to relevant tissues. DHA and EPA bound to phospholipids in KO have higher delivery efficiency than traditional fish oil and can be readily absorbed [9]. When compared to esterified n-3 PUFA in a randomized clinical trial, KO significantly improved the levels of high-density lipoprotein cholesterol, so-called good cholesterol, and apolipoprotein AI. Thus, it is more efficacious at reducing the levels of high-sensitivity C-reactive protein [10]. The effect of KO on disease activity index (DAI), colon length, and histological combined score (HCS) has been investigated using a rat UC model [11]. While KO marginally improved HCS, colon length was significantly preserved after KO supplementation. Moreover, in vitro data show that KO may have the potential to restore epithelial cell-cell adhesion and to improve mucosal healing [12]. A mixture of KO, probiotic *Lactobacillus reuteri*, and vitamin D has been shown to significantly improve clinical and histological scores, restore epithelial restitution, and reduce proinflammatory cytokines levels in dextran sulfate sodium (DSS)-induced colitis in mice [12] and has a modulatory effect on gut commensal bacteria. However, the molecular mechanisms that KO regulates the gut microbiome and microbe-derived metabolites remain largely unknown.

Chronic inflammation, which likely results from the disruption of pro-resolving pathways [13], is a hallmark of IBD. Intestinal macrophages are known to play an important role during the resolution of inflammation [14, 15]. In helminth-mediated T helper (Th) 2 models, alternatively activated or M2 macrophages [16] are polarized to facilitate tissue repair by inhibiting classically activated macrophages and elevating arginase-1 production. However, excessive M2 macrophage activation may impair intestinal protection against enteric bacterial infection and can aggravate intestinal injury [17]. Infection of pigs with the whipworm, *Trichuris suis*, induces a protective Th2 immune response and decreases the production of proinflammatory cytokines [4, 18]. Notably, the anti-inflammatory properties of *T*. *suis* have been exploited as a complementary therapy in IBD with some success [19–21]. In this study, we investigated the effect of KO on the attenuation of intestinal inflammation and the promotion of the appropriate resolution of inflammation and subsequent mucosal healing, a key therapeutic objective in the management of IBD, in both in vitro and porcine *T*. *suis* models using multi-omics approaches. We identified molecular and microbial signatures with high predictive accuracy for indicators of colitis pathophysiology. Furthermore, we validated some key findings using a *Citrobacter rodentium* inducing Th1-dependent colitis model in mice.

## Results

### Krill oil attenuated inflammation by modulating a broad range of signaling pathways in vitro

**Fig. 1:**
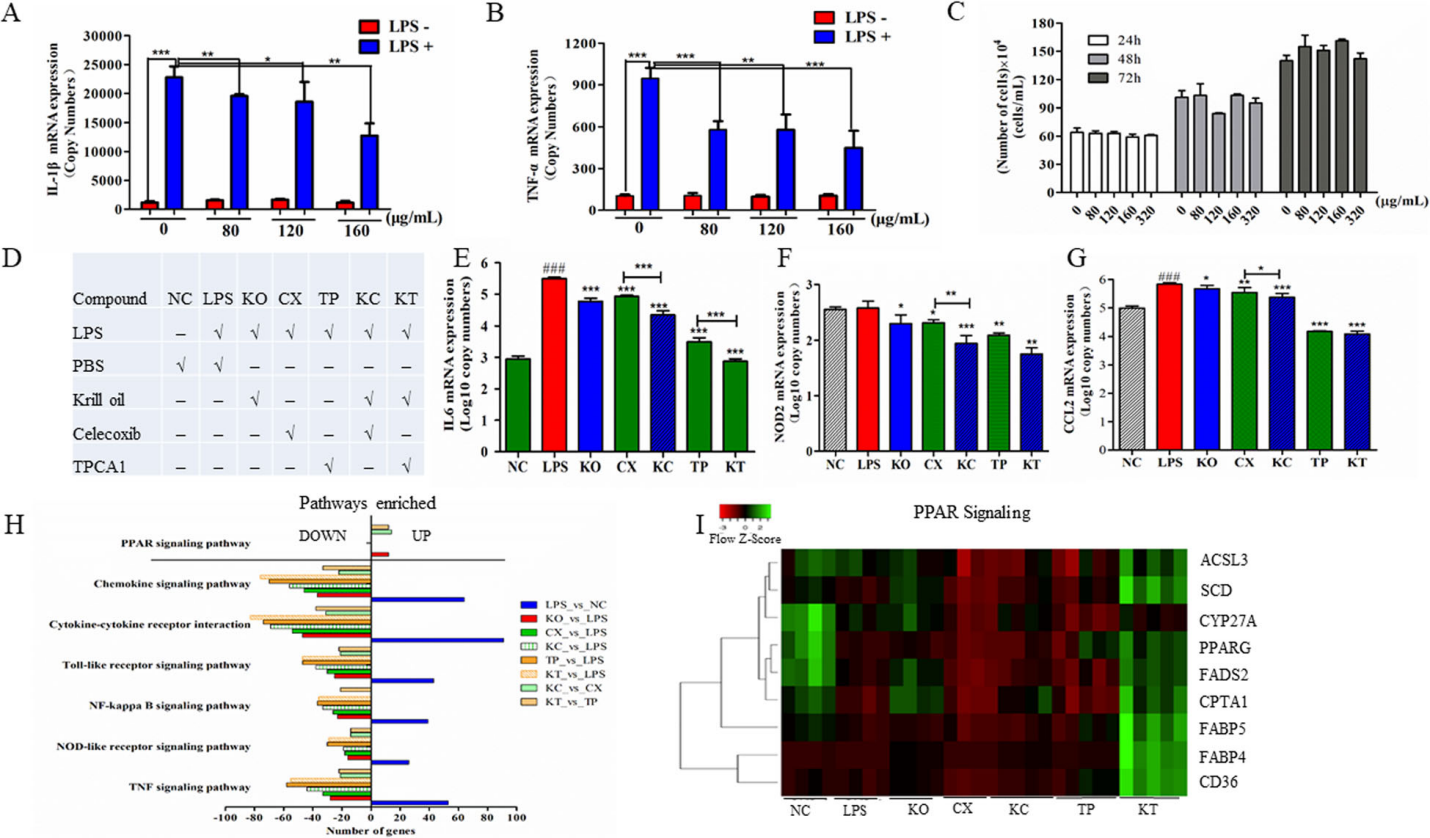
The effect of krill oil (KO), alone or in combinations with COX2 and IKK2 inhibitors, on pro-inflammatory cytokines and the transcriptome in human differentiated THP-1 cells treated with LPS. KO decreased LPS-induced mRNA expression of IL1β (**a**) and TNFα (**b**) in a dose-dependent manner. **c** The number of viable cells at various KO dose levels incubated for 24, 48, and 72 h. No cytotoxicity became evident at a dose up to 320 μg/ml. **d** Sample labels. KO displayed a synergistic effect in inhibiting inflammation mediators, such as IL6 (**e**), NOD2 (**f**), and CCL2 (**g**) at 160 μg/ml. **h** Pathways significantly enriched in differentially expressed genes detected using RNAseq transcriptome analysis. **i** A heat map showing genes in peroxisome proliferator-activated receptor (PPAR) signaling pathways regulated by KO and the inhibitors of COX and IKK2, alone or in combinations. ***p < 0.001; **p < 0.01; *p < 0.05. ^###^*p* < 0.001 (LPS vs. NC)

Treatment of differentiated THP1 human macrophages with KO significantly decreased lipopolysaccharides (LPS)-induced IL1β and TNFα mRNA expression in a dose-dependent manner (Fig. 1a, b). No cytotoxicity was detected at a dose up to 320 μg/ml of KO after a 72-h incubation (Fig. 1c). Approximately 53% reduction in LPS-induced IL1β and TNFα mRNA levels could be achieved with 160 μg/ml KO (*p* < 0.01). The synergistic effect of KO with two anti-inflammatory compounds, celecoxib (COX2 inhibitor, CX) and TPCA1 (IKK2 inhibitor), was investigated using RNAseq-based transcriptome analysis. Treatment of differentiated THP1 cells with LPS, TPCA1, or KO induced unique transcriptomes as indicated by the tight clustering of each group distinct from each other and the control group in a PCA plot (Supplementary Fig. S1 and S2). In contrast, CX clustered near KO suggesting that CX may be inducing similar transcriptomic changes as KO. Furthermore, KO-TPCA1 also clustered near KO and CX and was quite separated from TPCA1 suggesting that treatment with KO had a more dominant influence on the transcriptome than TPCA1. KO inhibited the expression of both COX1 and COX2 (FDR < 0.05), which likely provided a partial explanation of the observed transcriptome patterns between CX and KO. Moreover, KO in combination with either CX or TPCA1 resulted in a further reduction over KO alone in the expression of pro-inflammatory genes, such as IL6, NOD2, and CCL2 (Fig. 1b–g).

**Fig. 2:**
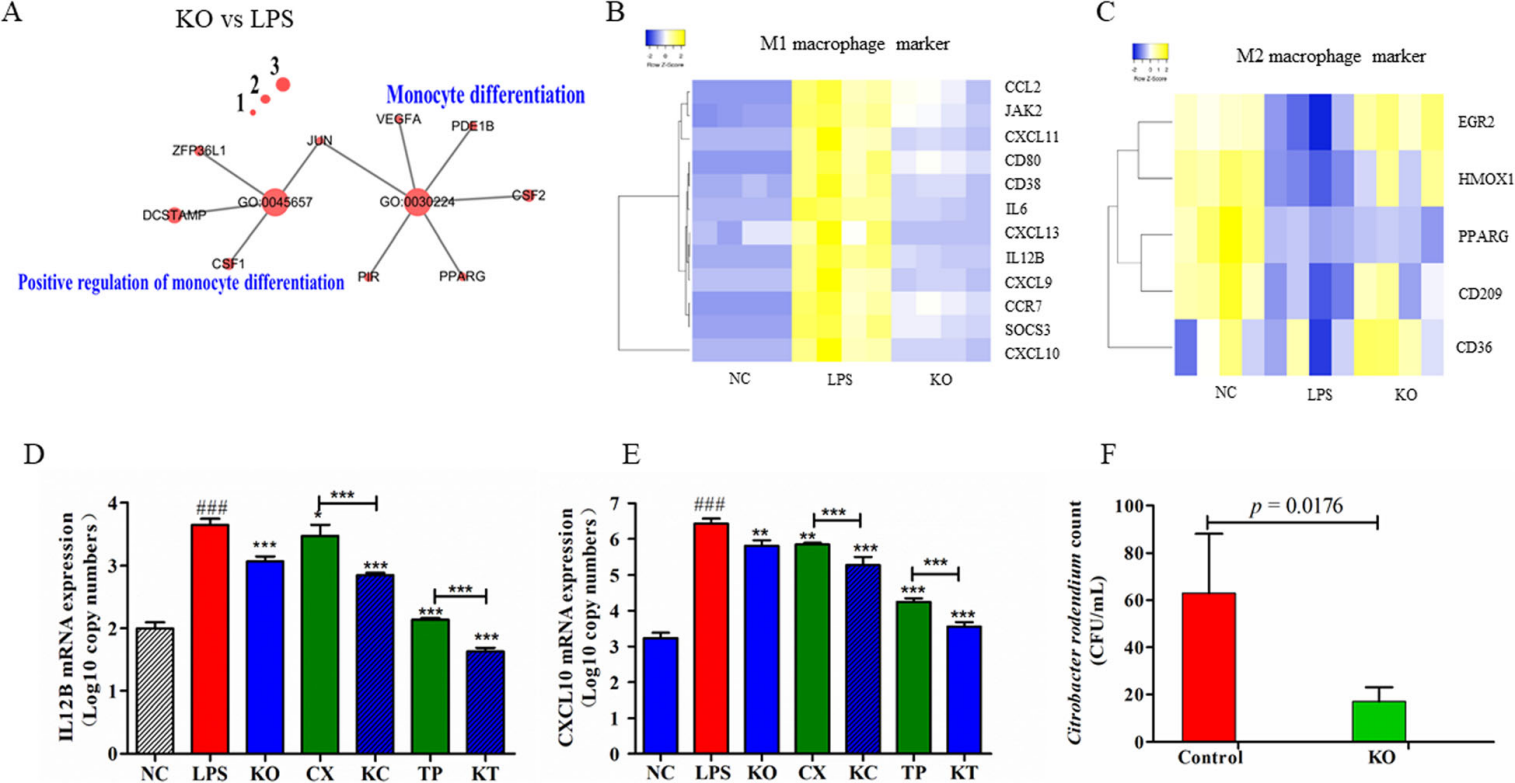
Krill oil (KO) enhanced intracellular bacterial killing in macrophages and regulated the expression of various macrophage marker genes in vitro. **a** Genes and Gene Ontology (GO) terms related to monocyte differentiation. **b** KO reversed the effect of LPS on M1 macrophage related marker genes. **c** M2 macrophage activation related genes were upregulated by KO. **d** LPS-induced expression of IL12B (**d**) and CXCL10 (**e**) was significantly inhibited by KO and COX2 and IKK2 inhibitors, alone or in combination. **f** KO enhanced intracellular bacterial killing of *Citrobacter rodentium* as the number of the bacteria surviving the gentamicin protection assay was significantly reduced. ***p < 0.001; **p < 0.01; ^###^*p* < 0.001 (LPS vs. NC)

KO, alone or in combination with celecoxib and TPCA1, inhibited multiple LPS-activated pathways in human macrophages. Among the downregulated genes, signaling pathways, such as cytokine-cytokine receptor interactions, NF-κB, and chemokine (Fig. S3A) as well as Nod-like receptor (Fig. S3B), Toll-like receptor, and TNF signaling, were significantly enriched (FDR < 0.001; Fig. 1h). Multiple genes involved in the peroxisome proliferator-activated receptor (PPAR) signaling pathway were significantly decreased by LPS (FDR < 0.05) but upregulated by KO, alone or in combination with celecoxib and TPCA1 (Fig. 1i). For example, KO reversed the effect of LPS-induced downregulation of PPARG and fatty acid-binding protein 5 (FABP5), a gene important in linking metabolic and inflammatory pathways (Fig. S4). KO also significantly inhibited the expression of IL17 receptor A (Fig. S5). Moreover, while LPS upregulated multiple M1 macrophage marker genes, such as CCL2, IL12B, CXCL9, CXCL10, CXCL11, and CD80, KO treatment for 48 h resulted in the reversal of the expression of these pro-inflammatory M1 genes (Fig. 2). However, KO restored the expression of LPS-inhibited M2 macrophage markers to the normal level (Fig. 2a–e). These findings suggest that KO may facilitate M1 to M2 polarization in human macrophages.

The effect of KO on macrophage phagocytosis and intracellular bacterial killing of THP1 human macrophages was also evaluated. The number of *C*. *rodentium* cells engulfed by macrophages was slightly increased in response to treatment with KO, compared to that in control untreated cells (Fig. S6). Moreover, the macrophage-mediated intracellular bacterial killing was significantly enhanced by KO (*p* = 0.0176) as the number of bacterial cells surviving the killing was markedly reduced (Fig. 2f). Together, our in vitro data provided evidence that KO had a modulatory effect in both the initial and pro-resolving phases of inflammation and that it may possess properties to promote mucosal healing.

### Krill oil mitigated intestinal mucosal damage in a Th2-driven porcine colitis model

**Fig. 3:**
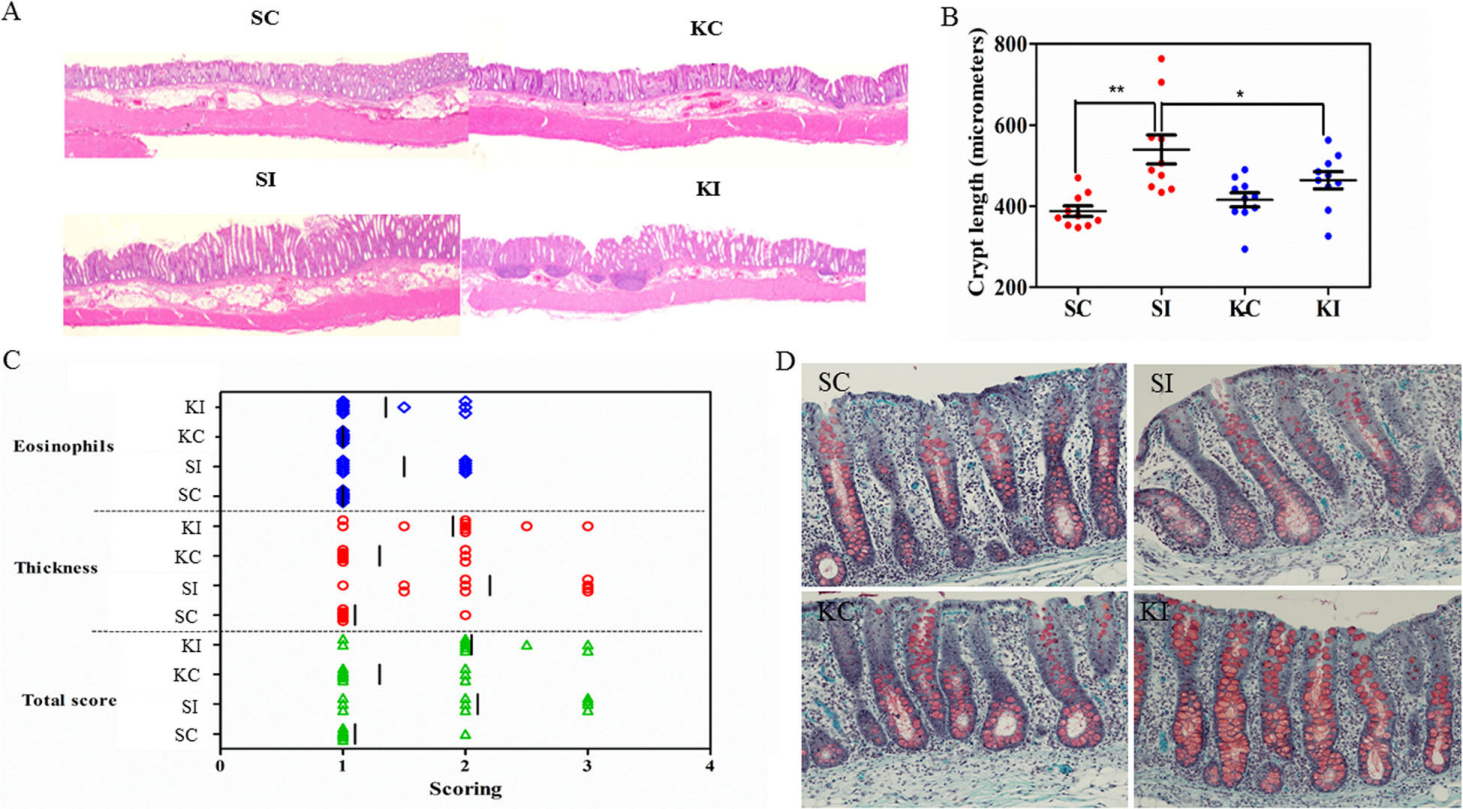
The effect of krill oil (KO) supplementation on the porcine colon tissue histological scores. SC: uninfected pigs fed soybean oil (SO). SI: infected pigs fed SO. KC: uninfected pigs fed KO. KI: infected pigs fed KO. **a** Gross morphology of the proximal colon. **b** Crypt length. **c** Total histological scores. **d** Goblet cells stained by Alcian blue and periodic acid-Schiff. **p < 0.01; *p < 0.05

A Th1/Th2/Th17 imbalance is an important driving force in the pathogenesis of colitis [22]. Recently, the Th2-like propensity of parasite excretory/secretory proteins has been exploited to ameliorate DSS-induced colitis via rebalancing the Th1/Th2 immune response [23]. The findings from our in vitro experiment which showed that KO inhibited Th1 immune responses and appeared to promote M1/M2 macrophage polarization inspired us to utilize a Th2-driven porcine colitis model to investigate anti-inflammatory properties of KO and its role in promoting the resolution of inflammation. Hematoxylin and eosin (H&E) staining shows that colon histological damage induced by *T*. *suis* infection was markedly improved by a 28-day KO supplementation (Fig. 3a). The infection resulted in a significant increase in crypt length, from 387.60 ± 40.86 in normal controls to 539.90 ± 113.38 μm (*p* = 0.0004). KO supplementation resulted in the partial reversal of the infection-induced increase in crypt length to a level observed in uninfected animals (464.00 ± 67.16, *p* = 0.043) (Fig. 3b). Colon smooth muscle thickness and overall histopathological scores were also marginally improved by KO (Fig. 3c). The number of goblet cells was notably increased by KO, particularly in infected animals (Fig. 3d). Further, there was a moderate reduction in *T*. *suis* larvae recovered from the cecum and colon of infected pigs fed KO (724.4 ± 263) versus those fed soybean oil (SO) as a control (861.3 ± 309). Underlying these morphological changes were the alterations in the tissue transcriptome. RNAseq analysis using the STAR-DESeq2 pipeline identified a total of 96 genes that were significantly affected by the infection in the proximal colon (Fig. S7A).

A weighted correlation network analysis (WGCNA) algorithm [24] was used to generate the consensus network (*N* = 40) and its KO and SO subnetworks (*N* = 20 per group). Among the 12 modules detected in the signed consensus network, the module turquoise was the largest with 11895 members, followed by the module grey (5074 genes). While the purple module (MEpurple) was smallest with 32 members, it was nevertheless significantly correlated with the gut histamine level (*p* = 7.0 × 10^−4^; Fig. S7B). No other modules in this network were significantly correlated with worm burden and fatty acid (22:6). In the SO subnetwork, the yellow module (MEyellow) was significantly correlated with gut histamine level (corr. = 0.67; *p* = 0.001) and worm counts (corr. = 0.52; *p* = 0.02). However, in the KO subnetwork, no modules were significantly correlated with any of the three physiological parameters. On the other hand, in the unsigned consensus network, at least two modules were significantly correlated with worm counts (data not shown).

In the purple module in the signed consensus network, the majority (> 90%) of module members were highly connected with very high eigengene-based module connectivity or module membership (kME > 0.85). These hub genes were likely critical components of the module and contributed to its overall function (Fig. S7C). The hub genes included ASB4, BANF2, IL1B, PAX7, RASGRP3, and TNR. Among them, PAX7 is a transcription factor (TF) with an important role in muscle development and homeostasis. We hypothesized that these hub genes may be co-regulated by common TFs.

At least two GO molecular functions, including high voltage-gated calcium channel activity (GO:0008331) and interleukin-1 receptor binding (GO:0005149), and 54 GO biological processes, such as regulation of lipid metabolic process (GO:0019216), positive regulation of vasculature development (GO:1904018), and positive regulation of icosanoid secretion (GO:0032305), were significantly enriched in the purple module (*p* < 0.01). Moreover, among the KEGG pathways enriched, the MAPK signaling pathway was most significantly overrepresented in the purple module (*p* = 2.50 × 10^−4^; FDR < 0.1; combined score = 187.45).

At least three TF binding sites significantly enriched in the purple module were detected using the TRANSFAC_and_JASPAR_PWMs function in the Enrichr pipeline [25]. For example, the potential binding sites for NR5A1 were significantly enriched in this module (*p* = 0.0022; Fig. S7D). Other two TFs, MZF1 5-13 and LTF, were also significantly enriched in the purple module (*p* < 0.05).

### Krill oil partially restored *Trichuris suis*-induced gut microbial dysbiosis

**Fig. 4:**
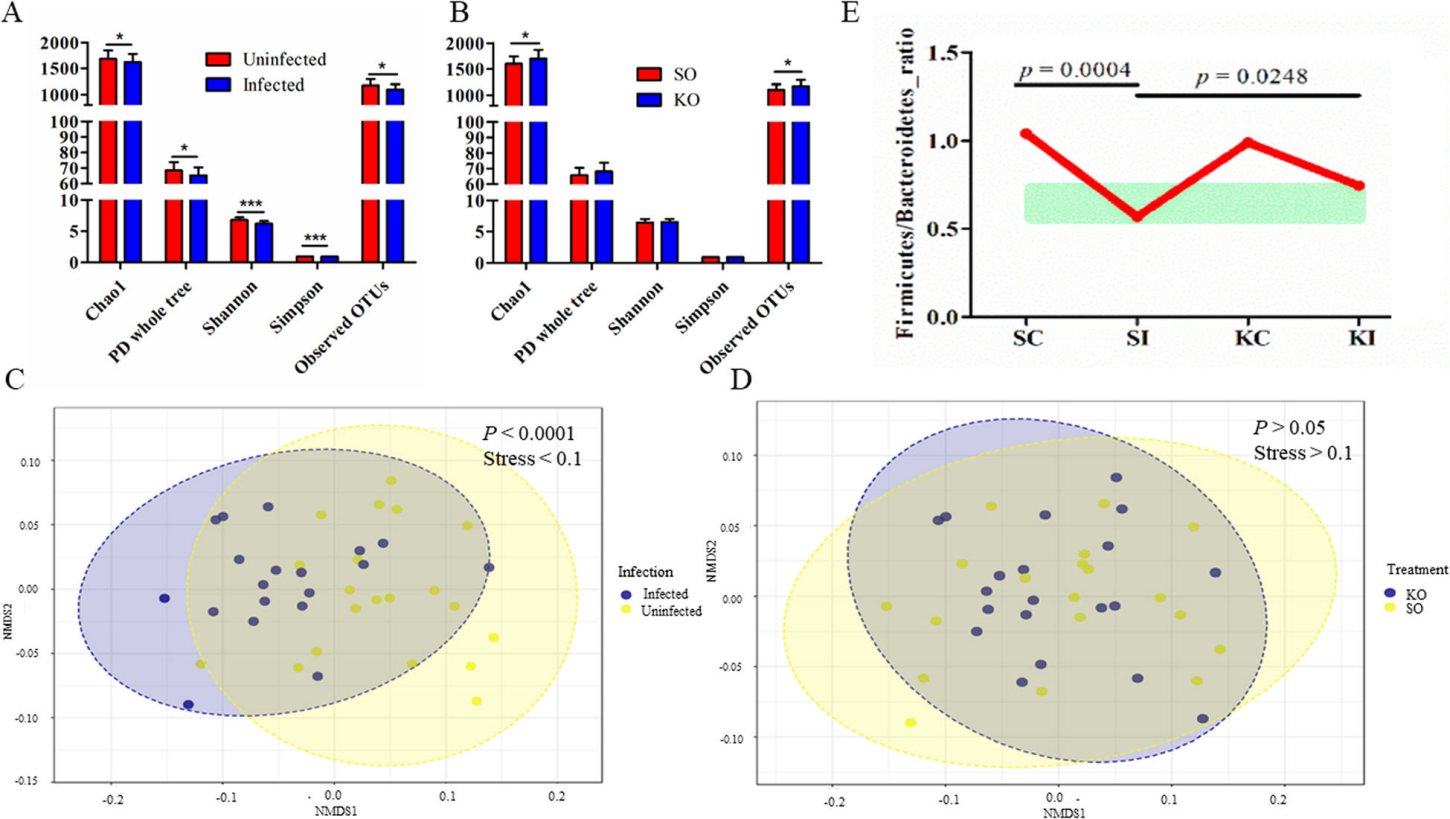
Krill oil modulated the gut microbiome in a porcine model. **a***Trichuris suis* infection in pigs had a significant effect on various microbial alpha diversity indices, compared to in uninfected pigs. **b** Krill oil (KO) supplementation increased microbial richness in the porcine proximal colon, with respect to control pigs fed soybean oil (SO). **c** The effect of the infection (**c**) and supplementation (**d**) on gut beta diversity as shown by non-metric multidimensional scaling (NMDS) based on a distance matrix derived from Jensen Shannon Divergence. **e** KO had a significant impact on *Firmicutes* to *Bacteroidetes* ratios in the proximal colon microbiome. SC: uninfected pigs fed SO. SI: infected pigs fed SO. KC: uninfected pigs fed KO. KI: infected pigs fed KO. ***p < 0.001; *p < 0.05

*Trichuris* infection significantly reduced various indices for alpha diversity in pigs (Fig. 4a), and feeding pigs with KO significantly increased two richness-based indices (Fig. 4b). For example, Chao 1 was increased from 1557.36 (± 242.78; SD) in pigs fed SO to 1702.13 (± 167.95) in pigs fed KO, regardless of the infection status (*p* = 0.0172). Phylogenetic diversity (PD) whole tree was also enhanced by KO from 63.86 to 68.23 (*p* = 0.0439). KO supplementation had no effect on species evenness, such as Shannon and Simpson indices. However, the infection had a more profound effect on gut microbial diversity, resulting in a significant reduction of the number of observed operational taxonomic units (OTU), PD whole tree, Shannon, and Simpson (*p* < 0.01, Fig. 4a). Unlike the dietary supplement, infection also had a significant impact on beta diversity (Fig. 4c). The results of permutational multivariate analysis of variance (PERMANOVA) suggest that up to 14.7% of the variance in the gut microbial composition can be explained by the effect of infection (permutation based *p* = 0.0001) while the KO factor only explained 2.4% of the variation. There were no significant interactions between infection and dietary supplementation. Non-metric dimensional scaling (NMDS) analysis based on Jensen Shannon divergence (Fig. 4c, d) also supported the hypothesis that the primary factor affecting the gut microbial composition and structure was the infection status, in agreement with the results obtained using analysis of similarities (ANOSIM, Fig. S8). Notably, feeding KO helped restore the microbial network structure in the infected condition (Fig. S9). While the number of the input OTUs for network construction from various groups were similar, *T*. *suis* infection decreased gut microbial interactions, as network nodes were reduced from 675 to 475 in response to infection in pigs fed SO. KO supplementation restored the number of nodes (654) and links (2025) to the normal level. One of the indicators of the infection-induced microbial dysbiosis was altered *Firmicutes* to *Bacteroidetes* (F/B) ratios. In the background of SO or KO supplementation, infection resulted in a significant decrease in the abundance of the phylum *Firmicutes* with a concomitant increase in that of *Bacteroidetes*. However, the infection-associated reduction in the F/B ratio was significantly improved by KO (Fig. 4e, *p* < 0.05).

**Fig. 5:**
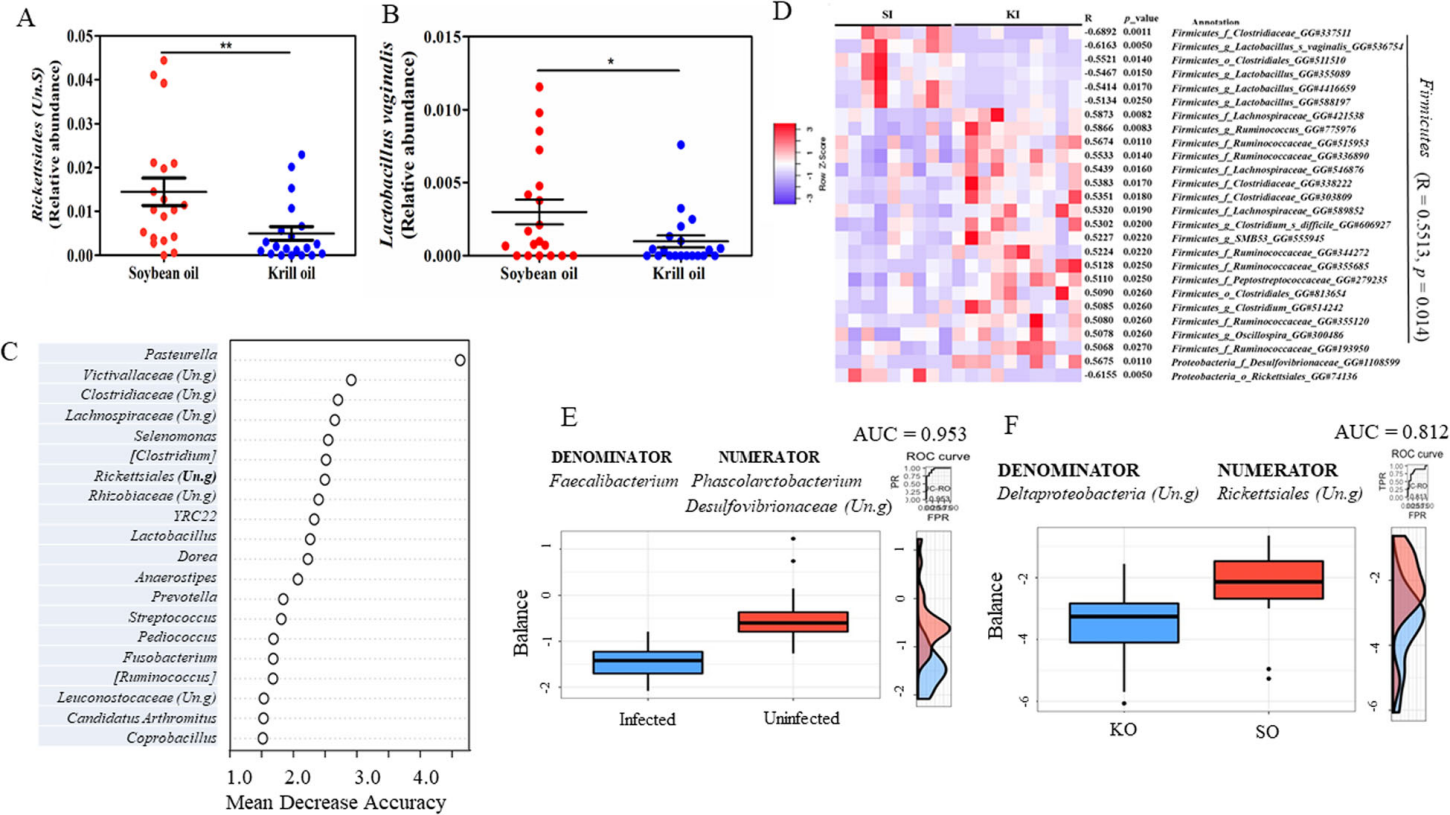
Important microbial taxa related to infection and supplementation status in pigs. **a** krill oil (KO) significantly decreased the abundance of an unclassified genus in the order *Rickettsiales* (**a**) and *Lactobacillus vaginalis* (**b**). **c** Select OTUs showing a significant correlation with KO supplementation in the infected condition. GG# represents Greengenes ID. **d** Important genera ranked by a random forest classification model contributed to classification accuracy with respect to the KO status. **e** Microbial signatures or global balances selected by selbal with a strong accuracy to distinguish the infection (**e**) and KO supplementation status (**f**). *AUC* area under the ROC (receiver operating characteristics) curve. The box plots represent the distribution of the balance values for each category. The right (vertical) panel of the figure represents the ROC curves with the AUC values (top) and density curves (bottom) for each category. **p < 0.01; *p < 0.05

Analysis of composition of microbiomes [26] (ANCOM) revealed a significant reduction in the relative abundance of an unclassified genus in the order *Rickettsiales* in response to KO supplementation (Fig. 5a). At the species level, the abundance of *Lactobacillus vaginalis* was also significantly decreased by ~ 3-fold in pigs fed KO (Fig. 5b). In addition, at least three other OTUs, Greengenes (GG) #355089 (*r* = -0.5467, *p* < 0.05), GG#4416659 (*r* = − 0.5414, *p* < 0.05), and GG#588197 (*r* = − 0.5134, *p* < 0.05), which were among the 26 OTUs that displayed a negative correlation with KO in the infected condition, were assigned to the genus *Lactobacillus* (Fig. 5c).

Machine learning algorithms, such as random forests (RF), are excellent tools for classifying microbiome features into various classes or categories, and which also allow us to dissect the relationships between microbial features and environmental attributes. RF is less sensitive to the sample size of the training data set and more accurate for prediction performance [27]. The 20 most important genera selected by RF provided an accuracy classification between the KO and SO groups (Fig. 5d). The genus *Pasteurella* was ranked among the most important features based on mean decrease accuracy (MDA) and was found to be 6.5-fold more abundant in KO than SO. *Lactobacillus* and an unclassified genus in *Rickettsiales*, the abundance of which was reduced in response to KO supplementation, were also among the most important genera in contributing to the classification accuracy. Notably, the abundance of segmented filamentous bacteria (SFB, previously *Candidatus Arthromitus*, and renamed as *Candidatus Savagella* [28]), which was 4.5-fold more abundant in KO fed pigs, was also one of the 20 important genera. *Faecalibacterium*, *Parabacteroides*, *Prevotella*, and *Ruminococcus* were among the most important genera with respect to classifying the infection status in the model (Table S2).

A microbial signature or balance with high accuracy in predicting the infected from uninfected groups was identified using selbal [29]. This balance, consisting of the two genera, *Phascolarctobacterium* and an unclassified genus in *Desulfovibrionaceae,* as a numerator and *Faecalibacterium* as a denominator, had an area under the receiver operating characteristic curve (AUC) of 0.953 (mean cross validation or CV-AUC = 0.78) for the infection status (Fig. 5e). A negative balance value in the infected group suggested that the two genera in the numerator had a much lower abundance than that in the denominator (*Faecalibacterium*). Moreover, a balance consisting of two genera in the phylum *Proteobacteri*a, including an unclassified genus in *Rickettsiales* (numerator) and an unclassified genus in *Deltaproteobacteria* (denominator), had high predictive accuracy for the KO supplementation (AUC = 0.812, Fig. 5f). The KO group had a much lower balance than SO, suggesting that the reduction in the relative abundance of the unclassified genus in *Rickettsiales* by KO played an important role in model performance and may have critical functional relevance. While considering infection as a covariant, a new balance, with the addition of two genera, *Cronobacter* and [*Clostridium*] as numerator and denominator, respectively, to the original two genera, resulted in an improved predictive accuracy for the dietary supplementation (AUC = 0.892).

### Inhibitory effects of krill oil on histidine metabolism contributed to its anti-inflammatory activities

Pigs fed a 4-week KO supplementation had a significant increase in serum EPA and DHA levels (Wilcoxon *p* < 0.001, Table S3). A slight but nevertheless significant increase in gut acetate concentration was observed in pigs fed KO (*p* < 0.05), leading to a marginal elevation in total short-chain fatty acid levels. Two balances with strong predictive power for both gut luminal acetate (Fig. S10A) and EPA values (Fig. S10B) were identified. Elevated EPA and DHA levels in the gut luminal contents of pigs fed KO were absorbed into the bloodstream, as indicated by a 2.5-fold increase in these PUFA in the serum (*p* < 0.001), compared to what was observed in pigs fed SO. Docosapentaenoate (ω-3 DPA) levels were also significantly elevated in the serum in response to KO; however, the serum level of pro-inflammatory ω-6 DPA (22:5n6) was significantly reduced in KO fed pigs (Table S3).

In the infected condition, 45 KEGG pathways were predicted to be significantly related with KO supplementation. Among them, the transcription factors pathway was positively correlated with KO (*r* = 0.8047, *p* < 0.001), while two pathways related to LPS, LPS biosynthesis and LPS biosynthesis proteins, were negatively correlated with KO (*r* < – 075, *p* < 0.001). Moreover, several pathways related to amino acids, such as histidine metabolism, tryptophan metabolism, and valine, leucine, and isoleucine biosynthesis, showed a significant divergence between the two dietary treatments (Fig. S11). They were negatively correlated with KO but positively correlated with SO.

**Fig. 6:**
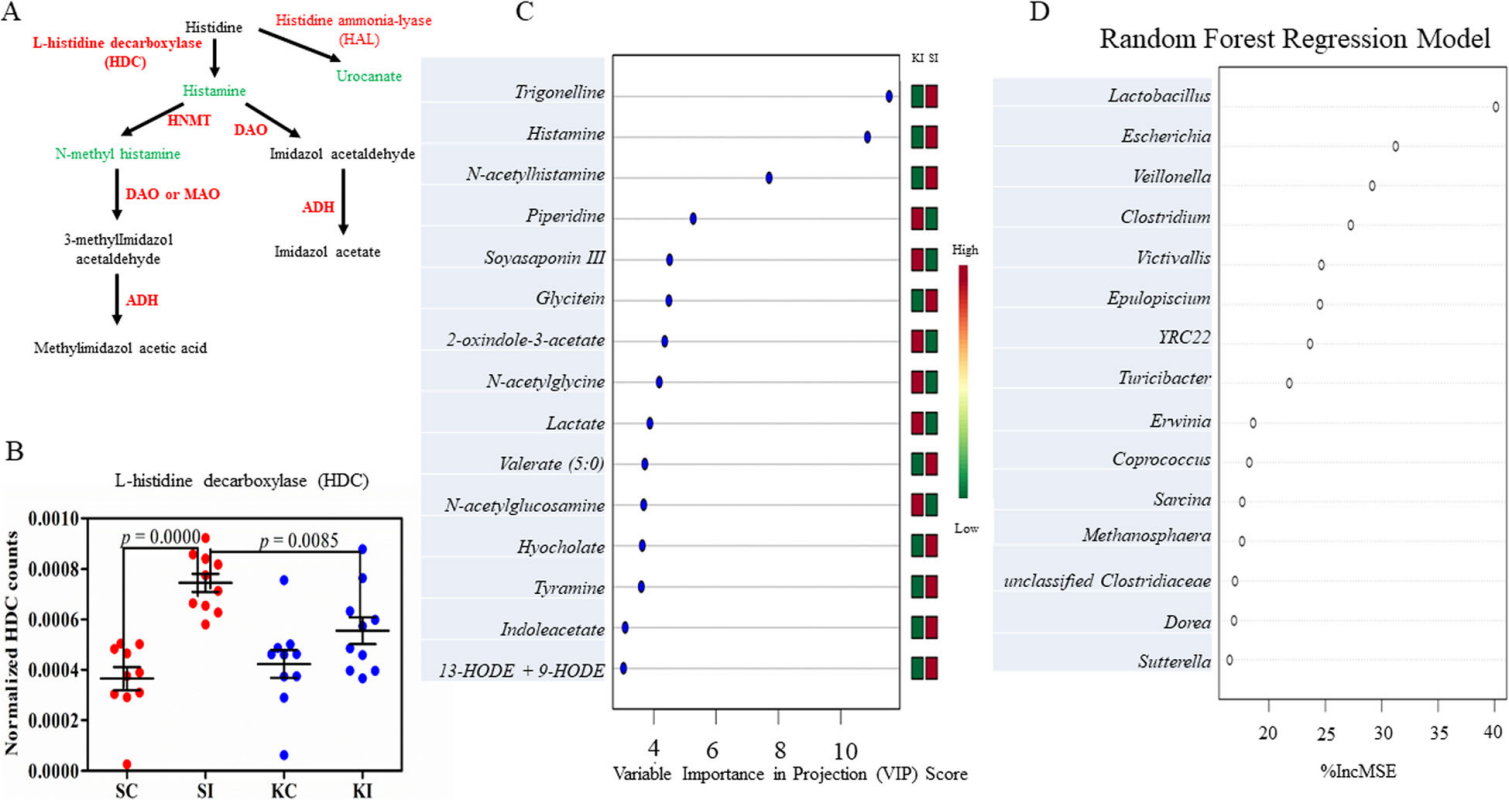
Krill oil inhibited histidine metabolism in pigs. **a** A diagram showing histidine metabolism pathway. **b** The normalized transcript abundance of a gene encoding l-histidine decarboxylase (HDC) that was significantly reduced by krill oil (KO) in proximal colon tissue. SC: uninfected pigs fed SO. SI: infected pigs fed SO. KC: uninfected pigs fed KO. KI: infected pigs fed KO. **c** The important metabolites in distinguishing the supplementation status ranked based on variable importance in projection (VIP) scores using partial least squares—discriminant analysis (PLS-DA) in infected pigs. **d** The important genera selected by the random forests regression model that were associated with colon luminal histamine levels. % IncMSE = % increase in mean squared error

The implication of the altered histidine metabolism pathway from the predicted data in the KO fed pigs prompted us to use untargeted metabolomics analysis to confirm the inhibitory effect of KO on histamine. As showed in Fig. 6a, histidine gets degraded into histamine and urocanate by l-histidine decarboxylase (HDC) and histidine ammonia-lyase (HAL), respectively. *T*. *suis* infection resulted in a 59.13-fold increase in gut luminal histamine concentration (*p* = 0.0216) and a 1.85-fold decrease in *cis*-urocanate (*p* = 0.0019), compared to the uninfected group (Table S4). Pigs fed KO had significantly reduced gut histamine from an elevated level induced by the infection. The increased luminal histamine by the infection likely resulted from the enhanced histamine biosynthesis of both host and microbial origins. The transcriptome analysis detected a 2.05-fold increase in HDC mRNA level (*p* < 0.0001) in the proximal colon tissue of infected pigs, compared to the uninfected control (Fig. 6b). More importantly, feeding KO resulted in a small but nevertheless significant reduction in the expression of HDC (Fig. 6b, *p* = 0.0085), a gene encoding the rate-limiting enzyme in histidine metabolism. Similarly, the levels of 1-methylhistamine and *N*-acetylhistamine were also significantly increased by infection (*p* < 0.05). Further, KO supplementation resulted in a 5- and 3-fold reduction in the concentration of these two metabolites, respectively, under the infected condition (Table S4, *p* < 0.05). Other metabolites in the pathway, such as 1-methyl-5-imidazoleacetate, 3-methylhistidine, 4-imidazoleacetate, 4-imidazoleacetate, imidazole propionate, and *N*-acetylhistidine, were also significantly affected by infection. However, KO did not appear to have a meaningful impact on their luminal concentration. The impact of the infection on histidine metabolism and subsequent modulation by KO appeared to be a localized event as untargeted metabolomics analysis in the serum samples of the same pigs did not detect any change in these metabolites (data not shown). This observation suggested that the modulatory effect of KO supplementation on inflammation was likely restricted to the colon mucosa.

Partial least squares discriminant analysis (PLS-DA) identified trigonelline, histamine, and *N*-acetylhistamine as the top 3 metabolites in distinguishing KO from SO under infection (Fig. 6c). To determine the impact of microbial community composition on the metabolites in histidine metabolism, a Pearson correlation analysis between the gut luminal concentrations of select metabolites and the relative abundance of various taxa was performed. Four genera were found to be significantly correlated with histamine and 1-methylhistamine (Fig. S12A, *r* > 0.56 and *p* < 0.0001). These four genera included *Megasphaera*, *Lactobacillus*, *Veillonella*, and an unclassified genus assigned to *Veillonellaceae*. The latter two are implicated in CD [30]. Two genera, *Desulfovibrio* and an unclassified genus in *Clostridiaceae*, displayed positive correlations with *cis*-urocanate (*r* = 0.63, *p* = 0.0000 and *r* = 0.57, *p* = 0.0002, respectively). Moreover, the correlation between *Lactobacillus* and 1-methylhistamine became even stronger among the infected pigs (*r* = 0.81, *p* = 0.0000). Further, at the species level, *Lactobacillus vaginalis* also showed a significantly positive correlation with histamine and 1-methylhistamine levels (Fig. S12B). The RF regression model suggested that there was a small but significant chance that gut histamine levels had any correlation with microbial taxa in the study, regardless of the infection status (*p* = 0.0150). The top microbial predictors for histamine levels identified by RF included *Lactobacillus*, *Escherichia*, *Veillonella*, and *Clostridium* (Fig. 6d). The correlation was improved somewhat in the infected condition. Similarly, the top predictors for 1-methylhistamine were *Clostridium*, *Lactobacillus*, *Turicibacter*, and *Escherichia* (data not shown).

**Fig. 7:**
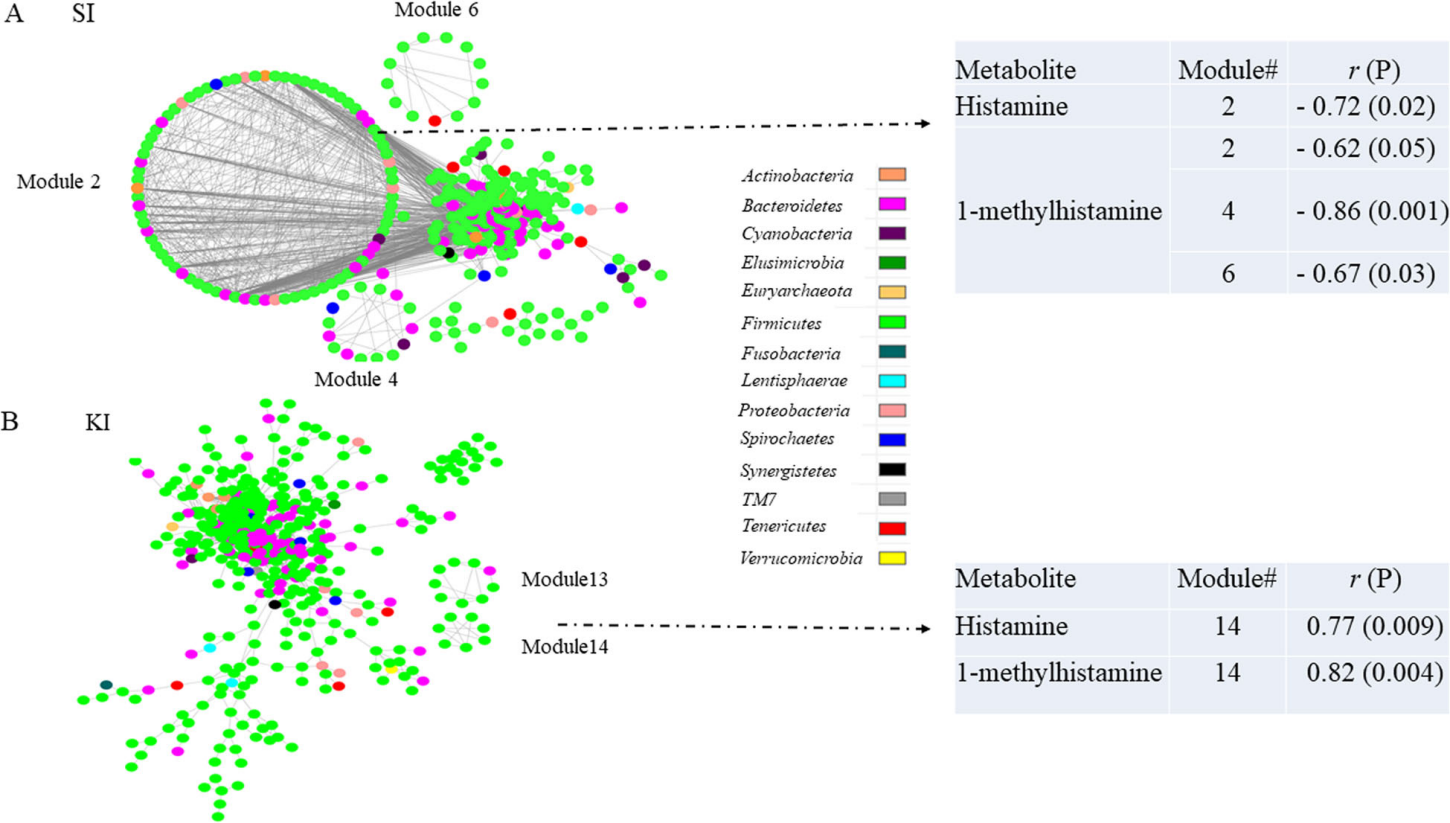
Network modules associated with gut luminal concentrations of histamine and 1-methylhistamine in the porcine model. SI: infected pigs fed soybean oil. KI: infected pigs fed krill oil. Solid line: positive correlation; dashed line: negative correlation. The color of each node (OTU) represents the phylum to which this OTU is assigned. *r*: correlation coefficient; P: significance (probability)

Network module-trait relationships were investigated using a Pearson correlation analysis. In the infected pigs fed SO, module #2 was negatively correlated with both histamine and 1-methylhistamine values (*p* < 0.05) while module #4 was negatively correlated with the latter (*r* = – 0.86, *p* = 0.001; Fig. 7a). A notable attribute of these two modules was that the majority of nodes (OTUs) belong to the order *Clostridiales*. In the network inferred from the infected pigs supplemented with KO, module #13 was negatively correlated with 1-methylhistamine (*r* = – 0.72, *p* = 0.0200). Eight of the nine nodes in this module belong to *Clostridiales*. Together, these findings suggested that strong microbial interactions of the species in the order *Clostridiales* may reduce gut 1-methylhistamine values. More intriguingly, module #14 (Fig. 7b) in the infected and KO supplemented network had a strong and positive correlation with both histamine (*r* = 0.77, *p* = 0.0090) and 1-methylhistamine (*r* = 0.82, *p* = 0.0040). Of note, all OTUs in module #14 belong to the genus *Lactobacillus*. While further experimental validation is still needed, these findings suggested that promoting strong microbial interactions using pre- and probiotics, among those species in *Lactobacillus* and *Clostridiales*, may have important modulatory effects on histidine metabolism.

### Validation of pro-resolving properties of krill oil and its modulatory effect on the gut microbiome using a murine Th1-dependent colitis model

**Fig. 8:**
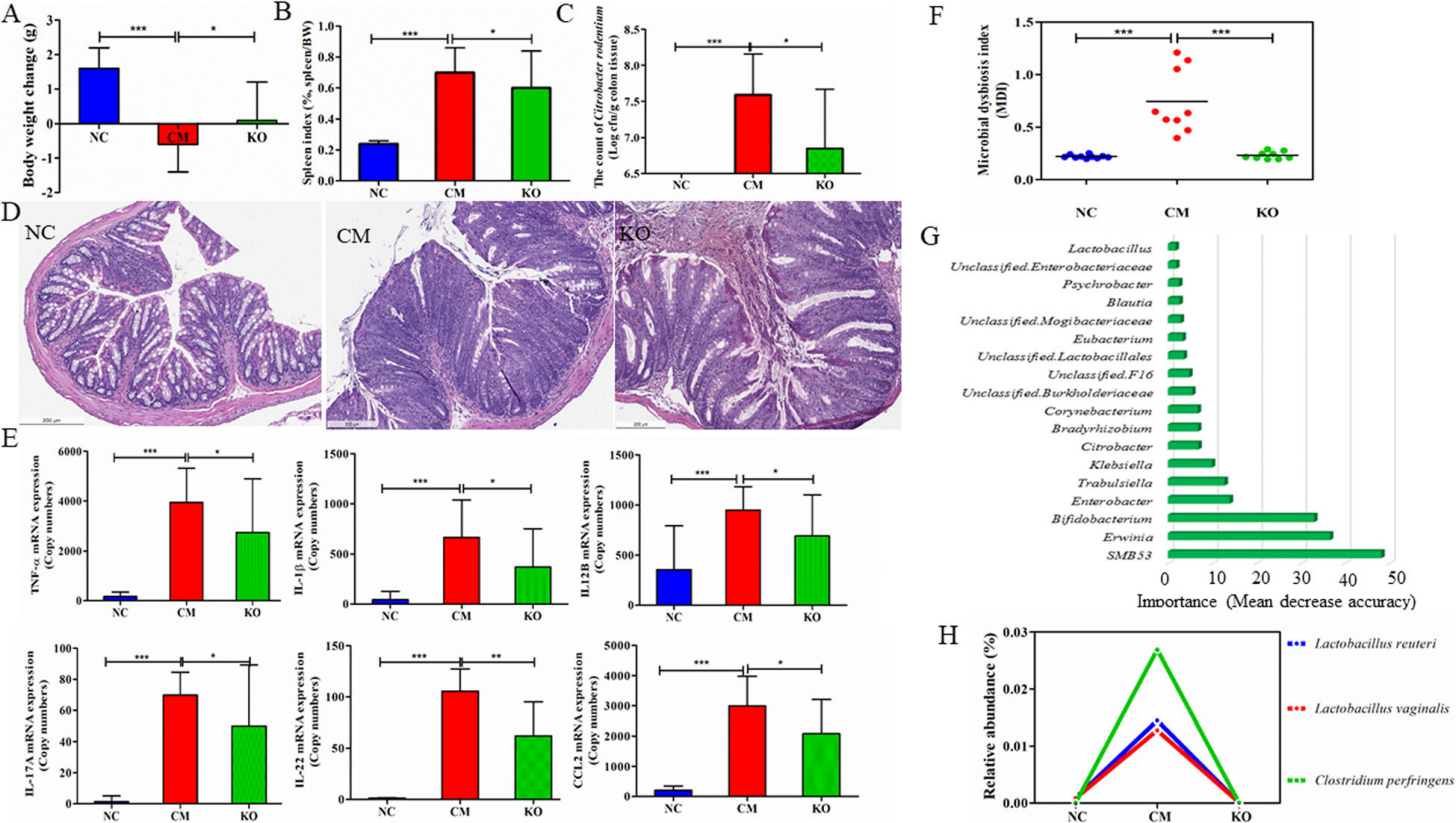
Krill oil supplementation mitigated *Citrobacter rodentium* induced colitis in mice. Krill oil (KO) improved bodyweight loss (**a**) and the spleen index (**b**), reduced the load of *C*. *rodentium* in the colon mucosa (**c**) and ameliorated histological scores (**d**). The expression of pro-inflammatory cytokines and chemokines, including IL1β, TNF, IL12B, IL17A, IL22, and CCL2 was significantly reduced by KO supplementation (**e**). **f** KO supplementation significantly improved the microbial dysbiosis index (MDI). *** Wilcoxon rank sum *p* < 0.001. **g** The 20 important genera selected by the random forests model for classification of the supplementation status (KO or PBS). **h** The relative abundance of three bacterial species, *Lactobacillus reuteri*, *L*. *vaginalis*, and *Clostridium perfringens* was reduced to the baseline level by KO supplementation. ***p < 0.001; **p < 0.01; *p < 0.05

To validate the importance and involvement of *Lactobacillus*-related species in regulating anti-inflammatory responses of KO, we used a *C*. *rodentium*-induced colitis model in C3H/HeNCr mice. Unlike Th2-inducing *T*. *suis*, *C*. *rodentium* induced a distinct Th1/Th17 immune response [17]. The mice infected with *C*. *rodentium* showed a significant loss in body weight and a notable increase in the spleen index (Fig. 8a, b, *p* < 0.05). The colon length and colon index, defined as colon weight in a 5.0-cm-long colon cylinder divided by total bodyweight, were also significantly affected by infection. Feeding KO partially reversed the body weight loss and improved the spleen index, compared to mice in the infected group (*p* < 0.05). The number of mucosa-attached *C*. *rodentium* bacterial load (per gram of colon tissue samples) was significantly decreased by KO, from 7.59 ± 0.57 (log colony forming units or cfu/g) in the infected group (CM) to 6.85 ± 0.82 (log cfu/g) (Fig. 8c, *p* < 0.05). The number of inflammatory infiltrates induced by *C*. *rodentium* was reduced in mice fed KO (Fig. 8d). Moreover, KO supplementation resulted in a significant decrease in the expression of pro-inflammatory cytokines, such as TNF, IL1β, IL12, IL17A, IL22, and CCL2, from a level elevated by *C*. *rodentium* infection (Fig. 8e).

Untargeted metabolome analysis identified at least ten metabolites with significantly reduced levels in the colitis mice than in healthy mice (Table 1, FDR < 0.05). These metabolites were involved in four pro-resolving pathways, such as aspirin-triggered resolvin E biosynthesis, aspirin-triggered lipoxin biosynthesis, leukotriene biosynthesis, and lipoxin biosynthesis. For example, the luminal concentration of resolvin E1 was 3.3-fold lower in the colitis mice while the levels of lipoxin A4 and B4 were 2.7-fold lower than those in healthy controls. KO supplementation significantly increased at least three metabolites involved in pro-resolving pathways, including aspirin-triggered resolvin E and lipoxin biosynthesis pathways. Together, these findings provided further support to the proposition that KO ameliorated both initial and pro-resolving phases of inflammation.

**Table 1:** Krill oil (KO) supplementation increased the metabolites involved in pro-resolving pathways, particularly the biosynthesis of E-series resolvins. Fold change was calculated based on normalized peak intensities in *Citrobacter rodentium* induced colitis mice (CM) divided by those in healthy control mice (Down dysregulation) or those in KO divided by CM (Up dysregulation). Pathway: A: aspirin triggered resolvin E biosynthesis; B, aspirin-triggered lipoxin biosynthesis; C, lipoxin biosynthesis; and D, leukotriene biosynthesis

Metabolite	Pathway*	Fold change	*P* value	Dysregulation	m/z	RT
(5S)-HPETE	D*	0.33	0.0004	Down	381.2270	3.974
(15R)-hydroxyeicosapentaenoate	B*, C**	0.36	0.0022	Down	365.2321	4.474
(5S)-HPETE	D*	0.40	0.0024	Down	395.2428	5.266
(5Z,8Z,11Z,14Z,17Z)-icosapentaenoate	A**	0.20	0.0037	Down	347.2217	7.754
15 [epi-] lipoxin A4/B4	B*, C**	0.37	0.0031	Down	411.2375	4.481
18R-hydroxy-eicosapentaenoate	A**, C**, D*	0.34	0.0020	Down	363.2167	4.736
5S hydro(peroxy),18R-hydroxy-eicosapentaenoate	A**	0.25	0.0009	Down	349.2010	5.702
Resolvin E1	A**	0.29	0.0023	Down	409.2217	3.840
15S-hydroxypentaenoate	B**, C***	1.70	0.0406	Up	340.2020	9.158
leukotriene A4	A*, B**, C***	1.68	0.0067	Up	363.2167	5.253
Resolvin E2	A*, B**, C***	1.79	0.0315	Up	315.1957	7.663

*m*/*z* mass-to-charge ratio, *RT* retention time in min

***p < 0.001; **p < 0.01; *p < 0.05.

*C*. *rodentium* infection resulted in a significant disruption in the gut microbial community. The infection significantly reduced both richness- and evenness-based α diversity, such as Chao1, Fisher’s α, and Shannon, as well as PD whole tree (*p* < 0.05, Fig. S13A). The infection also significantly impacted β diversity (Fig. S13B). NMDS based on a distance matrix derived from Jensen Shannon Divergence showed a clear separation between communities from the infected and uninfected mice (Fig. S13B). PERMANOVA results suggested that the infection alone explained 37.7% of the variation in the microbial community (Pseudo *F* = 10.28, permutation *p* = 0.0001). Feeding KO also had a significant effect on the beta diversity (Pseudo *F* = 1.94, *p* = 0.024). However, only approximately 10.8% of the variation in the microbial community could be attributed to the effect of KO. A 17-day KO supplementation did not appear to affect α diversity, possibly due to the short experimental duration.

*C*. *rodentium* infection had a profound impact on gut microbial composition. A total of 18 genera had altered abundance in the colitis mice as detected by ANCOM, compared to healthy controls. For example, the abundance of *Sutterella* was significantly increased by ~ 860-fold in the colitis model compared with healthy controls. A microbial dysbiosis index (MDI) was defined as an inverse log ratio of the sum abundance of *Coprococcus* and *Bacteroides* to the abundance of *SMB53*. As Fig. 8f shows, compared to healthy controls, the colitis mice had a significantly higher MDI (Wilcoxon *p* = 2.20 × 10^−5^). Feeding KO resulted in a significant improvement in MDI, from 0.74 in the colitis mice to 0.23 in mice fed KO (*p* = 4.10 × 10^−5^). KO also affected three of the 18 genera significantly altered by infection, *SMB53*, *Erwinia*, and *Bifidobacterium*. Notably, KO reversed the significant increase (~ 2800-fold) in the abundance of *SMB53* induced by infection to the baseline level. All three genera were the most important features contributing to the accuracy in classifying KO from the phosphate-buffered saline (PBS) group by RF (Fig. 8g). Both RF and sPLA-DA algorithms ranked *SMB53*, *Erwinia*, and *Bifidobacterium*, in this order, as the top three important features between KO and PBS groups. KO also had an important impact on the abundance of several OTUs related to *Lactobacillus.* The abundance of an OTU assigned to *Lactobacillus* was reversed to the baseline, from the > 2000-fold increase induced by infection. At least three bacterial species, *Clostridium perfringens*, *L*. *reuteri*, and *L*. *vaginalis* followed a similar trend (Fig. 8h). *Lactobacillus* and an unclassified genus in the order *Lactobacillales* were among the important features in KO classification by RF (Fig. 8g). Further, SFB were present in the gut of normal mice at a low abundance and became undetectable in the infected mice. As a result, *Candidatus Arthromitus* was ranked as one of the important features contributing to the classification accuracy between the *C*. *rodentium* infection status in mice (data not shown).

### Microbial signatures with high prediction accuracy for colitis-related pathophysiological traits in mice

**Fig. 9:**
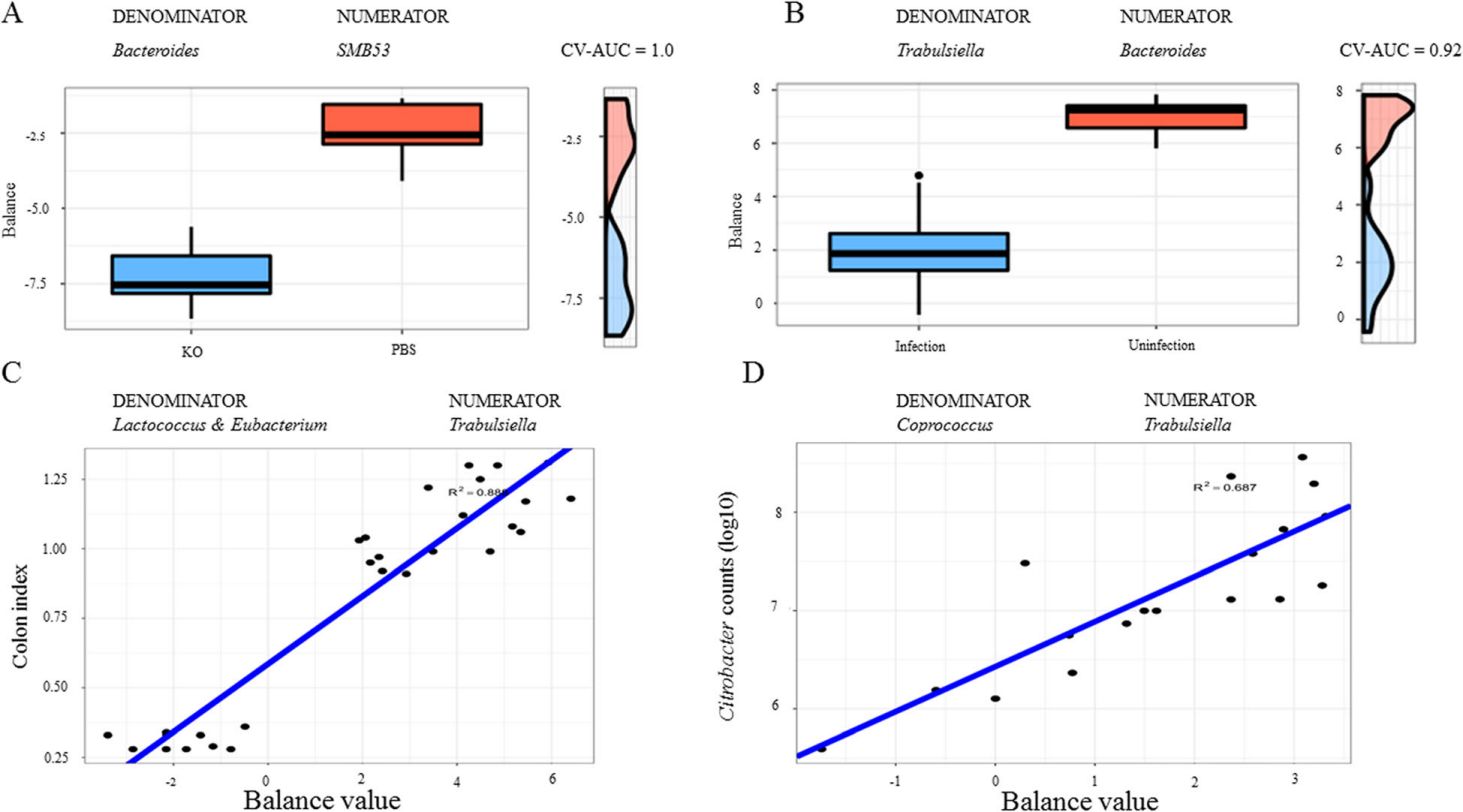
Microbial signatures or balances relevant to pathophysiological phenotypes of colitis in mice. Box plots represent the distribution of balance values in each category. The microbial taxa in the balance listed on the top of the chart. **a** The balance consisting of *SMB53* and *Bacteroides* discriminated KO from PBS controls. **b** The balance that distinguished the infection status is shown. The right sides of the charts represent the density curve for each category. CV-AUC = cross validation adjusted mean area under the ROC curve (AUC). The balances showing strong associations with colon index (**c**) and the *Citrobacter rodentium* bacterial load (counts) in the colon tissue (**d**). The *y*-axis represents the numerical values for the colon index (**c**) and log10 *Citrobacter* counts (**d**), respectively. The *x*-axis is the balance value. The lower balance values were associated with the lower colon index or reduced bacterial loads, or an improved colitis phenotype

*C*. *rodentium* infection and KO supplementation in mice had a marked effect on several colon pathophysiological parameters, such as colon length, colon index, spleen index, and *C*. *rodentium* counts per gram of the colon tissue. A global balance consisting of *SMB53* (numerator) and *Bacteroides* (denominator) accurately predicted KO supplementation from PBS controls (CV-AUC = 1.0, Fig. 9v). The balance values were negative, suggesting that the relative abundance of *SMB53* was much lower than that of *Bacteroides*. Lower balance values indicated that KO supplementation was associated with a significant reduction in the abundance of *SMB53* and a concurrent increase in that of *Bacteroides.* A microbial signature consisting of *Bacteroides* and *Trabulsiella* classified the microbial communities of the infected and uninfected mice with high accuracy (CV-AUC = 0.92, Fig. 9b). A microbial signature, consisting of *Odoribacter* (numerator) and an unclassified genus in *RF32* (denominator), had a strong predictive power for colon length (*R*^2^ = 0.864) while a microbial signature consisting of *Trabulsiella* and *Lactococcus* and *Eubaterium* had a strong predictive accuracy for the colon index (*R*^2^ = 0.888, Fig. 9c). Moreover, a microbial signature of *Trabulsiella* (numerator) and *Coprococcus* (denominator) strongly predicted *C*. *rodentium* counts in the colon (*R*^2^ = 0.687; Fig. 9d). Of note, there were two OTUs assigned to the genus *Citrobacter* while seven OTUs were assigned to the genus *Trabulsiella* (and four of them annotated to *T*. *farmeri*) using the Greengenes database in this study. The European Nucleotide Archive suggests that the Greengenes assignment of *Trabulsiella* may be incorrect [31]. The representative sequences of the OTU involved also mapped to *Citrobacter* in the Ribosomal Database Project database but at a 90% similarity level. Together, these data suggest that the genus *Trabulsiella* identified in the mouse model may be due to *Citrobacter*.

## Discussion

Dietary supplements rich in PUFA, such as fish oil, have been widely used in the management of various diseases, including colitis. In recent years, consuming KO has gained popularity due to its advantages over traditional fish oil [32, 33]. Rich in ω-3 PUFA (up to 31.5%), particularly in readily absorbed phospholipid forms, KO is known to have improved bioavailability. Moreover, KO contains a potent antioxidant, astaxanthin, up to 873.0 mg per kg, and has the potential to reduce oxidative stress [11]. In this study, we demonstrated that KO possesses strong anti-inflammatory activities by modulating a broad range of signaling pathways, including the NF-κB and NOD signaling pathways and inhibits pro-inflammatory cytokines in vitro. The findings are in a good agreement with published reports that KO exerts its inhibitory effect at the initial phase or onset of inflammation [11].

Dysregulation of the pro-resolving phase of inflammation, particularly lipid mediators, is associated with chronic inflammation, a hallmark of colitis. KO appears to be involved in the resolution phase of inflammation as evidenced by its ability to promote M2 polarization and enhance macrophage intracellular killing in vitro. In the mouse colitis model, KO significantly increased the gut luminal levels of several metabolites, such as resolvin E2, related to pro-resolving pathways, particularly aspirin-triggered biosynthesis of resolvin E and lipoxins. Multiple metabolites in these pathways were dysregulated in colitis models, compared to healthy controls. Resolvins are known to orchestrate the timely resolution of inflammation [34]. In addition to their direct anti-inflammatory effect, lipoxins and resolvins also promote the resolution of inflammation by enhancing macrophage-mediated clearance of apoptotic neutrophils [35, 36]. For example, resolvin E2 regulates neutrophil chemotaxis and enhances phagocytosis and anti-inflammatory cytokine production [37]. Future work will examine the correlation between the variability in PUFA intake and metabolism among individuals, luminal resolvin levels, and improvements in colitis severity. Moreover, KO displayed a synergistic effect with COX2 and IKK2 inhibitors in suppressing pro-inflammatory mediators induced by LPS in THP1 cells. Its potential to mitigate helminth parasite induced intestinal tissue damage and subsequently promote mucosal healing was observed in a porcine model.

In this study, we demonstrated that KO has a strong modulatory effect on the gut microbiome in both porcine and murine models. KO significantly improved the gut microbial dysbiosis index and increased microbial richness, which corroborated previous findings that n-3 PUFA increases microbial diversity in middle aged and elderly women [38]. Current therapeutics for colitis-related diseases have been heavily focused on targeting the initial phase of inflammation. A shift to focus on the resolution phase of inflammation, especially using diet-based disease modifiers, may provide a better alternative in the management of IBD. Based on the knowledge obtained from our study, it is likely that standard therapies using corticosteroids, immunosuppressants, or biologicals in combination with long-term KO intake in IBD patients may yield extra health benefits. The full benefit of long-term KO intake may result from concerted actions of multiple metabolic and signaling pathways.

Histamine is an important immunomodulator and can exert pleiotropic effects via interactions with its receptors [39]. Histamine levels and histamine 4 receptor (H4R) expression in the mucosa of patients with active UC are significantly elevated [40]. Urinary excretion of *N*-methylhistamine is also significantly increased in IBD patients. Moreover, a significant correlation of *N*-methylhistamine excretion with clinical disease activity is established [41]. Significantly higher levels of urine histamine and methylhistamine have been detected in people with gastrointestinal food allergy under unrestricted diets [42]. Further, histamine drives severity of innate inflammation via H4R in experimental colitis models. In this study, we demonstrated that multiple key metabolites in the histidine metabolism pathway, such as histamine and 1-methyhistamine, were significantly reduced by KO supplementation from an elevated level induced by infection in colon luminal contents. Histamine was ranked among the top three variables in distinguishing KO from SO groups in the infected condition using a PLS-DA model (Fig. 6c). A concomitant increase in *cis*-urocanate level was also detected in the animals supplemented with KO. Together, these data suggest that KO likely affected histidine metabolism by tilting the balance of histidine conversion toward urocanate via HAL. Our findings that mRNA expression of HDC, a gene encoding the rate-limiting enzyme catalyzing histidine to histamine conversion, was significantly reduced in the porcine colon mucosa provided further support to this notion. Indeed, KO supplementation disrupted a module (MEyellow) that showed a significant correlation with gut histamine levels observed in the SO subnetwork, which may also have contributed to the reduced histamine levels from those SO fed animals. Moreover, the expression of all four histamine receptors was detected while the abundance of H4R was higher than that of H2R in the colon mucosa. In addition to the reduced biosynthesis by host cells, histamine of microbial origin is also important. Indeed, RF regression models identified *Lactobacillus*, *Escherichia*, *Veillonella*, and *Clostridium* as the most important features correlated with gut luminal histamine levels (Fig. 6d). Certain strains of *L*. *reuteri* contain a gene cluster encoding HDC [43] and convert dietary histidine to histamine, which in turn activates H2R and regulates acute inflammation [44]. Histamine from *L*. *reuteri* increases cAMP, which inhibits the downstream MEK/ERK MAPK signaling via protein kinase A and suppresses TNF production [45]. Our data showed that the abundance of *L*. *reuteri* as well as several other *Lactobacillus* species was significantly reduced by KO (Fig. 8h). It is still unclear if the *L. reuteri* strains impacted in this study harbor the HDC gene cluster. However, it is conceivable that KO may regulate histamine of microbial origin via suppressing *Lactobacillus* abundance. Together, our data suggest that the regulation of histidine metabolism may represent a previously unappreciated mechanism through which KO attenuates intestinal inflammation. The intimate interactions among KO, histamine and its receptors, and the gut microbiome should be further investigated. Mechanistic understanding of these interactions may hold promises for the development of novel alternative therapeutics.

As a modest inflammatory effector, IL17 acts concertedly with other inflammatory mediators, such as TNFα and IFNγ, on pathogenic and protective processes in autoimmune disease and cancer [46]. While IL17 is generally considered to be involved in IBD due to its role in repairing intestinal damage [47] and regulating gut permeability [48], anti-IL17 biologics failed to offer any protection in CD patients [49]. In our study, KO significantly decrease IL17RA expression in human macrophage-like THP1 cells (FDR adjusted *p* = 6.21 × 10^−6^) while repressing IL17A expression in mouse colon mucosa. Indeed, ω-3 PUFA precursor α-linolenic acid and derivatives (EPA and DHA) inhibit IL17A secretion by decreasing intercellular adhesion molecule 1 expression in human monocytes and adipose-derived stem cells, providing evidence for the beneficial effects of ω-3 PUFA in restraining IL17-related inflammation. In our porcine *T*. *suis* infection model, feeding KO to pigs resulted in a 4.5-fold increase in colon luminal incidence of SFB. As a result, SFB were one of the important features identified by RF in discriminating KO from SO (Fig. 5c). SFB are a crucial factor driving Th17 cell differentiation and inducing IgA production [50]. On the other hand, Th17 cells control SFB burden [51]. Substantial SFB overgrowth is observed in IL17RA knockout mice; also, anti-IL17RA treatment in wild-type mice increases SFB colonization [51]. In pigs, SFB are mainly attached to the ileal epithelium [30]. In our study, gut luminal contents were sampled from the proximal colon. It is unclear if the increased SFB abundance in luminal contents is related to the effect of KO on SFB attachment. Moreover, SFB are known to play a protective role against *C*. *rodentium* infection in mice. SFB colonization reduces the capacity of inoculated *C*. *rodentium* to grow and invade colonic tissues possibly via the action of Th17 cytokines, such as IL22 [50]. Controlling the number of SFB to colonize the ileum can alter the course of Th17 cell-related disease and protective immunity against bacterial infection. In future work, we will focus on understanding the mechanistic connection among KO supplementation, IL17 signaling, and SFB colonization using better defined animal models.

Microbial signatures, a group of taxa that can better predict treatment outcomes or a phenotype of interest, have pragmatic utilities. Balances or log ratios of relative abundances among groups of taxa can overcome the problem of differences in sample size and be developed as biomarkers [29]. In this study, we identified multiple microbial signatures that have high discriminative power or predictive accuracy for dietary treatment effects or are strongly associated with colitis-related pathophysiological phenotypes. In the porcine model, a balance consisting of two unclassified genera in *Proteobacteria*, one in *Rickettsiales* and another in *Deltaproteobacteria*, had a relatively high discriminative power for the KO supplementation. The order *Rickettsiales* includes a group of obligate intracellular bacteria that are common parasites of eukaryotes and zoonotic pathogens [52]. The abundance of *Rickettsiales* was significantly reduced by feeding KO (Fig. 5a). Lower (negative) balance values were discriminative for KO. Together, inhibitory effects of KO on these zoonotic pathogens may add some extra health benefits. Feeding KO significantly increased EPA and DHA in both gut luminal contents and the serum. A microbial signature, consisting of the genus *CF231* (numerator) and two genera, *vadin CA11* and *Dehalobacterium* (denominator), had a strong association with gut luminal EPA levels. *Dehalobacterium* is known to have a negative association with body mass index [53].

In the murine *C*. *rodentium*-induced colitis model, the abundance of at least 18 genera was significantly increased, compared to healthy controls. For example, the abundance of *Erwinia* and *Sutterella* was > 500-fold higher while that of *Ruminococcus* and *Coprococcus* was significantly lower in colitis mice, consistent with previous findings in CD patients [54]. Moreover, several predictive balances for colitis-related phenotypes were identified. A balance consisting of *Odoribacter* and an unclassified genus in *RF32* had a high predictive accuracy for colon length (*R*^2^ = 0.864). *Odoribacter* has been shown to have a decreased abundance in pediatric CD patients, compared to healthy controls [55]. Similarly, two microbial signatures, *Trabulsiella* and *Sutterella* and an unclassified genus in *S24-7* and *Trabulsiella* and *Lactococcus* and *Eubaterium*, were strongly predictive for colon weight (*R*^2^ = 0.922) and colon index (*R*^2^ = 0.888), respectively. *Sutterella* is known to be implicated in several diseases, including IBD [56] and autism [57]. The direct link between increased *Sutterella* abundance and IBD may involve the gut-brain axis [58]. The role of *Sutterella* in the pathogenesis of colitis should be examined in future studies. While the relative abundance of a single taxon may be less relevant, balances consisting of a group of taxa can provide a better discrimination for colitis-related phenotypes, and thus may serve as a valuable biomarker for colitis severity.

## Conclusions

We examined the effect of KO supplementation on both the initial and pro-resolving phases of inflammation. KO inhibited the expression of Th1 and Th17-related cytokines and promoted the bactericidal activities of macrophages in vitro. KO partially restored microbial dysbiosis in the models of infection-induced colitis by increasing species richness and modulating microbial interactions. Further, the inhibitory effects of KO on key metabolites in histidine metabolism of both host and microbial origin contributed to its anti-inflammatory activities. Future direction will include the exploration of synergistic effect of KO with conventional small molecule drugs with respect to promoting the proper resolution of intestinal inflammation.

## Methods

### Krill oil

Krill oil (KO) samples were provided by Jedwards International (Braintree, MA, USA). The lot used in this study contained 41.0% total phospholipids. EPA and DHA contents were 23.3% and 13.4%, respectively. Moreover, KO contained a potent antioxidant, astaxanthin, at the concentration of approximately 873.0 mg/kg. The detailed KO composition analysis is listed in Table S1.

### Cell culture

THP1 cells, an immortalized monocyte-like cell line derived from the peripheral blood of a child with acute monocytic leukemia, were obtained from ATCC (Manassas, VA, USA) and used for in vitro experiments. THP1 cells were differentiated using phorbol 12-myristate 13-acetate (PMA) as a model for human macrophages [59]. Briefly, THP1 monocytes were grown at 37 °C in 5% CO_2_ with RPMI-1640 medium (ATCC) supplemented with 10% fetal bovine serum and 50 U/ml of penicillin and 50 μg/ml of streptomycin (ThermoFisher, Waltham, MA, USA). Cells were seeded onto T175 flasks at a density of 5 × 10^5^ cells per ml and differentiated with PMA at 25 ng/ml for 48 h with a daily medium change. After PMA differentiation, KO cytotoxicity was determined by a Trypan blue dye exclusion assay at various dose levels, up to 320 μg/ml for 72 h. KO emulsion was prepared according to a published protocol [60] and used for all subsequent experiments. The anti-inflammatory activities of KO and other inhibitors, alone or in combinations, were evaluated on PMA differentiated THP1 cells. The compounds were added to the media for a total of 48 h (fresh compounds were replaced every 24 h) as follows: KO at 160 μg/ml, 20 μM celecoxib (Sigma, St Louis, MO, USA), 1 μM TPCA1 (TP, Abcam, Cambridge, MA, USA), KO + celecoxib (KC), and KO + TPCA1 (KT), respectively. After 42-h incubation, LPS (Sigma) was added to the media at a final concentration of 10 ng/ml for 6 h. The equal volume of PBS was used for controls (NC). The assays were conducted with four to five replicates. The media were saved for ELISA assays, and macrophages were harvested for total RNA isolation.

The effect of KO on macrophage phagocytic and bactericidal activities was evaluated using Gentamicin protection assay [61]. PMA-differentiated THP1 cells were incubated in the media containing either KO (160 μg/ml) or PBS (negative controls) for 48 h. The cells were then carefully washed with antibiotic-free media and infected with *C*. *rodentium* at a desired multiplicity of infection of 10:1 at 37 °C for 60 min (T0). The *C*. *rodentium* culture is described below. After infection, cells were washed three times with cold PBS and incubated with gentamicin containing medium (100 mg/ml) for 2 h (T1). Gentamicin kills extracellular bacteria that are not engulfed by macrophages while intracellular bacteria engulfed are not affected by this antibiotic. The cells at each time point were washed with sterile PBS three times and then lysed immediately in 0.2 ml sterile macrophage lysis buffer. The lysates were mixed with 0.8 ml sterile PBS; and serial dilutions were plated and counted after overnight incubation at 37 °C. The assays were repeated four times. The percentage of intracellular bacterial killing was calculated [61].

### *Citrobacter rodentium* culture

A nalidixic acid-resistant *C*. *rodentium* strain DBS100 (ATCC# 51459) was used in the experiment as previously described [62]. A frozen stock of *C*. *rodentium* was streaked out on a lysogeny broth (LB) agar plate and used to inoculate LB media and incubated overnight at 37 °C with shaking. The culture was expanded and grown to an OD600 of ~ 1.5. The bacteria were collected by centrifugation and then resuspended in LB at a concentration of 1.25 × 10^10^ cfu per ml the following morning. The dose was later confirmed by retrospective plating. The *C*. *rodentium* load in the tissue was determined by plating on LB agar plates with 50 μg/ml nalidixic acid for selection.

### Animals and diets

Forty pigs (White Yorkshire x Landrace; 9–10 weeks of age, mean bodyweight: 17.9 kg) were purchased from Oak Hill Genetics (Ewing, IL, USA). The pigs were raised with free access to food and water and randomized into four groups using a 2 × 2 factorial design with the parasitic whipworm *T*. *suis* infection as the first factor and the dietary treatment as the second factor. The four groups (*N* = 10 per group) were (1) uninfected and (2) infected fed SO; and (3) uninfected and (4) infected fed KO. The 20 infected pigs received a single oral dose of 5000 infective *T*. *suis* eggs while the 20 uninfected pigs received PBS. The infection progressed for 21 days after inoculation. SO and KO dietary treatments started 7 days prior to the infective egg inoculation and lasted for a total of 28 days. Pigs received a single daily dose of 1.5 g of either SO or KO individually mixed in a freshly made sugar-coated cookie dough ball in the morning. Assuming the mean human bodyweight is 60 kg, 1.5 g of daily ingestion of KO in this study is a human equivalent dose of 5.0 g.

For the validation experiment, 30 5-week-old C3H/HeNCr male mice were acquired from Charles River (Frederick, MD, USA) and fed a basal AIN-93M diet throughout the entire experimental duration (Research Diets, New Brunswick, NJ, USA). Two weeks after arrival, the mice were randomly divided into three groups (*N* = 10 per group): uninfected and supplemented with PBS (NC), *C*. *rodentium* infected and supplemented with PBS (CM), and *C*. *rodentium* infected and supplemented with KO. After acclimation, the mice in the CM and KO groups were infected with *C*. *rodentium* at a single dose of 2.5 × 10^9^ cfu in 0.2 ml PBS by oral gavage. Uninfected mice received 0.2 ml of sterile PBS by oral gavage. The infection was allowed to progress for 12 days after inoculation. The mice in NC and CM groups received a daily dose of 0.2 ml PBS by oral gavage while the mice in the KO group received 0.2 ml KO in water emulsion (1.5 mg KO per mice per day) via oral gavage 5 days prior to the infection, which continued until the end of the experiment (i.e., a total of 17 days including 12 days after inoculation). Mice were monitored and weighed daily. Mice that lost more than 25% of their body weight and/or became moribund were euthanized. Feces were collected at various time points post-inoculation; also, the amount of *C*. *rodentium* secreted in feces was monitored. Mice were weighed and euthanized on day 12 post-infection. The spleen tissue was aseptically removed, weighed, homogenized in LB, and plated on LB agar plates with no antibiotic to enumerate the total bacterial load. The entire colon was excised and the length measured. The entire colon contents including fecal pellets were collected, mixed, and snap frozen in liquid nitrogen for metabolite profiling and microbiome analysis. The emptied colon was weighed and subdivided into 1-cm portions. One section was fixed in 10% neutral-buffered formalin (NBF, Sigma) for histology and one snap was frozen in liquid nitrogen for total RNA extraction and subsequent RNAseq transcriptome analysis. The colon section adjacent to the anus was homogenized in LB for bacterial load determination and expressed as log10 cfu/g of colon tissue.

### Tissue histology

Approximately 1 cm proximal colon tissue was fixed in 10% NBF and sectioned at 5-μm thickness for H&E staining. Goblet cells were stained using Alcian Blue and periodic acid-Schiff (PAS). Surface epithelial cells (0–4), edema (0–2), hemorrhage (0–2), crypt dilation (0–2), thickness of smooth muscle layer (0–3), and the number of inflammatory infiltrates (0–3) were scored based on a previously published system [63].

### Gene expression analysis using qRT-PCR and RNAseq

Total RNA was extracted using Trizol reagents (Invitrogen, Carlsbad, CA, USA) according to manufacturer’s instructions. Crude total RNA was further purified using a QIAGEN RNeasy Micro Kit with DNase digestion to remove possible genomic DNA contamination. The RNA concentration was measured using a Nanodrop ND-1000 spectrophotometer (Thermo Scientific, Wilmington, MA, USA). RNA integrity was verified using a BioAnalyzer 2100 (Agilent, Palo Alto, CA, USA).

For qRT-PCR, cDNA was synthesized from total RNA using an iScript Advanced cDNA Synthesis Kit (BioRad, Hercules, CA, USA). Quantitative qRT-PCR reactions were carried out in a CFX Connect Real-Time PCR Detection System (BioRad). The reactions were run in duplicates in a total volume of 22 μl containing the following: 2 μl of cDNA (100 ng), 0.5 μl of each primer (forward and reverse, 20 nM each), 11 μl of SsoAdvanced Universal SYBR Green Supermix (BioRad), and 8 μl of nuclease-free water. The amplification reactions were subjected to an initial denaturation at 95 °C for 3 min, followed by 40 cycles of 95 °C for 30 s, 60 °C for 30 s, and 72 °C for 30 s. A standard-curve-based absolute quantification method was used [64].

RNAseq libraries were prepared using an Illumina TruSeq RNA sample prep kit (Illumina, San Diego, CA, USA) following the manufacturer’s instructions. The libraries for each sample were pooled at an equal molar ratio and based on their respective sample-specific barcodes. Paired-end sequences were generated at 51 bp/read using an Illumina NextSeq 500 sequencer.

The quality of raw sequences was checked using FastQC (Babraham Institute, Cambridge, UK). Raw sequences were then trimmed using Trimmomatic (v0.38). The preprocessed reads were analyzed using both Hisat2-String Tie-DESeq2 [65] and STAR-DESeq2 pipelines [66] with default parameters. FDR < 0.05 was used as a cutoff for determining differentially expressed genes. Gene enrichment analysis, including Gene ontology (GO) and the Kyoto Encyclopedia of Genes and Genomes (KEGG) pathway assignment, was conducted using the Database for Annotation, Visualization and Integrated Discovery [67] (DAVID v6.8).

The WGCNA R package (v1.69) was used to generate co-expression networks for the porcine transcriptome dataset. The *goodSamplesGenes* function was applied to filter samples and genes with too many missing values and those with zero variance. The signed network was derived based on a biweight midcorrelation (bicor) method. The soft threshold power, R-squared, threshold was set to 0.85. The minimum module size was 30. The module preservation was calculated using the *modulePreservation* function in the package. Both network-based composite preservation statistics, *Zsummary* and *medianRank*, were derived. Empirical thresholds, Zsummary < 10 or medianRank > 8 as originally proposed [68], was used as the cutoff for non-preserved modules. The hub genes were defined as those with absolute module membership (*k*ME) values ≥ 0.95. Functional enrichment analysis, such as the pathway and gene ontology (GO) function of the select modules, was conducted using the Enrichr package [25]. Furthermore, the TRANSFAC_and_JASPAR_PWMs section of the EnrichR algorithm was used to identify potential common transcription factors (TF) with *p* < 0.05 as a cutoff for significance.

### 16S rRNA gene sequencing

ZymoBIOMICS Microbial Community Standard (Cat# D6300) was obtained from Zymo Research (Irvine, CA, USA) as a microbiome standard. In addition, nuclease-free water was used as a non-DNA template (negative) control (NDT). The standard consists of three Gram-negative and five Gram-positive bacteria and two yeasts with a defined composition. Both NDT and the standard were processed along with experimental samples following the same protocol and parameters to validate the entire workflow, from total DNA extraction and sequencing to data analysis.

Total DNA was extracted from colon contents as previously described [69]. Briefly, a QIAamp Fast DNA Stool Mini Kit (QIAGEN, Germantown, MD, USA) was used with some modifications. First, a bead-beating bacterial cell wall disruption procedure using a FastPrep 5G instrument and Lysing Matrix E (MP Biomedicals, Irvine, CA, USA) was added. Second, lysis at 70 °C was extended to 8 min.

The hypervariable V3–V4 regions of the 16S rRNA gene amplification and sequencing were performed [64]. The primer sequences were as follows: forward primer, 341/357F, CCTACGGGNGGCWGCAG; reverse primer, 805/785R: GACTACHVGGGTATCTAATCC. A total of 20 cycles of PCR amplification was conducted. The amplified products from individual samples were purified using Agencourt AMPure XP beads (Beckman Coulter, Danvers, MA, USA). The purified PCR products were quantified using BioAnalyzer 2100 DNA 7500 chips and pooled based on an equal molar ratio and their respective samples-specific barcodes. The pooled libraries were sequenced using an Illumina MiSeq Reagent Kit v3 (2 × 255 cycles) as described previously [70].

### Bioinformatic analysis of 16S rRNA gene sequences

Feature or OTU tables were generated using the Quantitative Insights Into Microbial Ecology QIIME1 (v.1.9.1) [71] and QIIME2 (v 2019.07) pipelines [72].

The quality of raw reads was checked using FastQC v0.11.2. The sequences with low-quality score and the four maximally degenerate bases (NNNN) at the most 5′ end of the primer were removed using Trimmomatic v0.38. The paired end reads were merged using join_paired_ends.py with the following parameter settings: the minimum overlap length was 20 bp and the maximum allowed mismatches within the overlapping region was 5%. The command pick_closed_reference_otus.py was used for OTU picking. Taxonomy assignment was based on the Greengenes database (v13.8). *Alpha_diversity.py* command line was used for alpha-diversity index extraction at an OTU level. Several tools, such as NMDS, principal coordinate analysis (PCoA), PERMANOVA, and ANOSIM, were used for beta-diversity analysis based on various distance matrices. PICRUSt (v1.1.2) was used to predict metagenome functional contents from 16S rRNA marker gene survey data with default parameters based on the OTU table generated using the closed-reference protocol [73].

QIIME2: all reads were pooled using the *qiime tools import* script. The *qiime dada2 denoise-paired* was used for sequence quality control. The subsequent procedures were conducted as described [72].

Microbial co-occurrence networks were constructed using a random matrix theory (RMT)-based pipeline with default parameters as described [74]. OTU detected in > 50% of the samples were retained for network construction. A fast-greedy modularity optimization procedure was used for module separation. The within-module degree (*Z*) and among-module connectivity (*P*) were calculated and plotted to generate a scatter plot for each network. The module-environmental trait relationships were analyzed using Pearson correlation coefficients. The network was visualized using Cytoscape v3.6.1.

For the detection of taxa differing significantly in two or more populations or experimental groups, a novel statistical framework based on a statistic W, ANCOM (v2), was used [26]. The algorithm was designed to address compositionality issues inherent in the marker gene count data.

Microbial signatures or balances were identified using selbal (R version 3.6.1) with default parameters [29]. The randomForest R package (v4.6-14) was used. Both RF classification and regression models were performed in the study. The OTU table generated from QIIME1 was first collapsed to a genus level count table. The abundance data were then transformed based on total sum scaling. The RF parameters used were as follows: the number of trees in the forest (ntree) was set to 501 and the number of features randomly sampled at each node in a tree (mtry) was 13. The Z-score, or scaled mean decrease accuracy, was calculated and used to rank feature or variable importance.

### Metabolomic analysis

Short-, medium-, and long-chain fatty acids in gut colon contents were determined using 1290 Infinity II liquid chromatography (LC) Systems (Agilent) coupled to a Sciex 4000 QTRAP mass spectrometer (MS) with an electrospray ion source (ESI). Lipids were analyzed in the multiple-reaction monitoring mode with a negative ion detection. Each sample (100 mg) was carefully weighed, and 1.0 ml of methanol and 5-mm I.D. stainless metal balls was added to the sample and homogenized using an Mixer Mill MM 400 (Retsch, Haan, Germany). Homogenized samples were sonicated at 4 °C for 5 min followed by centrifugation at 21,000 g for 15 min. The supernatant collected (50 μl) was mixed with 25 μl of 200 mM 3-nitrophenylhydrazine (3NPH) and 25 μl of 150 mM 1-ethyl-3-(3-dimethylaminopropyl) carbodiimide (EDC)–HCl–6% HPLC grade pyridine solution according to a previously reported procedure [75]. These chemicals were obtained from Sigma. After a 40-min reaction at 30 °C, 400 μl of 60% methanol and 50 μl of an internal standard containing the 3NPH derivatives of fatty acid standards were added to the sample. After mixing, 5 μl was injected onto a Waters BEH C18 column for LC separation and subsequent MS analysis. Concentrations of fatty acids in each sample were calculated from internal standards and expressed as nmol per gram of gut contents.

Untargeted metabolomics analysis of gut contents was conducted as described [76]. Briefly, individual samples were accurately weighed. Methanol was added to precipitate proteins. After removing organic solvents, processed samples were characterized by an Ultra-performance liquid chromatography (UPLC)–tandem mass spectrometer (UPLC-MS/MS), consisting of an Acquity UPLC System (Waters, Milford, MA, USA) and a Q Exactive high resolution/accurate mass spectrometer (Thermo Scientific) interfaced with a heated ESI source. Raw data were extracted, peaks were identified, and then quantified using the area under the curve method. Each compound was corrected in run/day blocks. The peak intensity data were normalized based on the median and log transformed. Normalized data were analyzed using a modified *t* test or Wilcoxon rank sum test to identify metabolites that may differ significantly among experimental groups. In addition, raw spectral data were analyzed using the XCMS pipeline with default parameters [77].

## Supplementary information

**Additional file 1: Figure S1.** A PCA plot showing that krill oil (KO), COX2 inhibitor (celecoxib) and IKK2 inhibitor (TPCA1) induced distinct transcriptome patterns in human THP1 cells differentiated using phorbol 12-myristate 13-acetate (PMA). **Figure S2.** MA plots (log ratio vs mean average) for the visualization of differences in global transcriptome features of THP1 cells under different treatment conditions. The percentage denotes the number of up (red) or down (green) regulated genes induced by various compounds at a false discovery rate (FDR) < 0.05. **Figure S3.** Heatmaps showing genes in Chemokine (A) and Nod-like receptor signaling (B) pathways significantly inhibited by krill oil and other inhibitors at FDR < 0.05. **Figure S4.** Krill oil acted synergistically with COX2 and IKK2 inhibitors in increasing the expression of PPARG and FABP5 in human THP1 cells differentiated using PMA. **Figure S5.** The expression of IL17RA was significantly inhibited by krill oil in human THP1 cells *in vitro*. **Figure S6.** The number of bacteria engulfed by human macrophage phagocytosis was marginally increased by krill oil *in vitro*. **Figure S7.** A. A heat map showing the genes displaying a significant difference in abundance in the four experimental groups in the proximal colon tissue in pigs in response to *Trichuris suis* infection and dietary supplements. SC: uninfected pigs fed SO. SI: infected pigs fed SO. KC: uninfected pigs fed KO. KI: infected pigs fed KO. B. Modules-trait relationships in the signed consensus network. The correlation between pathophysiological traits, worm count, gut histamine levels, and gut fatty acid (FA_22:6) measurements, and the module eigengene value was calculated based on Pearson correlation. C. A scatterplot showing gene significance (*y*-axis) vs. module membership (*x*-axis) in the purple module. D. Transcription factors significantly enriched in the purple module in the signed consensus network. **Figure S8.** ANOSIM analysis of beta diversity in the porcine proximal colon microbiome. **Figure S9.** Global microbial interaction networks inferred using a Random Matrix Theory (RMT) based algorithm in the porcine proximal colon microbial community. **Figure S10.** Microbial signatures or balances associated with acetate and eicosapentaenoic acid (EPA) concentrations in proximal colon contents in pigs. **Figure S11.** Select pathways significantly correlated with soybean (SO) and krill oil (KO) supplementation. **Figure S12.** Taxa significantly correlated with gut luminal concentrations of histamine, 1-methyhistamine, and/or cir-urocanate in a *Citrobacter rodentium* induced murine colitis model. **Figure S13.***Citrobacter rodentium* infection in mice had a significant impact on gut microbial diversity. **Table S1.** Composition analysis of krill oil and soybean oil used in the study. **Table S2.** Top 20 genera selected by Random Forests that distinguish the infection status in a porcine model. **Table S3.** Serum long chain polyunsaturated fatty acid (LCFA) in pigs. KO: krill oil. SO: soybean oil. HMDB: The Human Metabolome Database. **Table S4.** The metabolites related to Histidine Metabolism was significantly affected by krill oil supplement (KO) in pigs infected by *Trichuris suis*.

## Data Availability

All raw sequence data were deposited to NCBI SRA database with free public access. The following are the accession numbers: Human THP1 transcriptome RNAseq data: PRJNA601651; Porcine proximal colon tissue transcriptome data: PRJNA601460; Porcine proximal colon content 16S rRNA gene sequences: PRJNA601338; Mouse gut 16S rRNA gene sequences: PRJNA601328; Microbiome Standard and NDT: PRJNA601657. In addition, the raw data for untargeted metabolome analysis of porcine proximal colon contents are freely accessible at the Mendeley data (10.17632/p832v28fcc.1). The raw spectral data for mouse metabolome analysis are freely available at 10.17632/p832v28fcc.1. The feature and OTU tables can be downloaded at 10.17632/v9vczp77tb.1.

## Abbreviations

ANCOM: Analysis of composition of microbiomes;
ANOSIM: Analysis of similarities
AUC: Area under the receiver operating characteristic curve
DHA: Docosahexaenoic acid
EPA: Dicosapentaenoic acid
FDR: False discovery rate
HDC: l-histidine decarboxylase
IBD: Inflammatory bowel disease
IKBKB: Inhibitor of nuclear factor kappa B kinase subunit beta or IKK2
IL: Interleukin
KO: Krill oil
LPS: Lipopolysaccharides
NMDS: Non-metric dimensional scaling
OTU: Operational taxonomic units
PBS: Phosphate-buffered saline
PCoA: Principal coordinate analysis
PERMANOVA: Permutational multivariate analysis of variance
PPAR: Peroxisome proliferator-activated receptors
PUFA: Polyunsaturated fatty acids
SO: Soybean oil
Th: T helper
