# Supplementary material for: Mechanistic insights into the attenuation of intestinal inflammation and modulation of the gut microbiome by krill oil using in vitro and in vivo models
Source: Microbiome. 2020 Jun 4;8:83. doi: 10.1186/s40168-020-00843-8 (PMC7273679; doi:10.1186/s40168-020-00843-8)
Supplement: Supplementary file 2 — Additional file 1: Figure S1. A PCA plot showing that krill oil (KO), COX2 inhibitor (celecoxib) and IKK2 inhibitor (TPCA1) induced distinct transcriptome patterns in human THP1 cells differentiated using phorbol 12-myristate 13-acetate (PMA). Figure S2. MA plots (log ratio vs mean average) for the visualization of differences in global transcriptome features of THP1 cells under different treatment conditions. The percentage denotes the number of up (red) or down (green) regulated genes induced by various compounds at a false discovery rate (FDR) < 0.05. Figure S3. Heatmaps showing genes in Chemokine (A) and Nod-like receptor signaling (B) pathways significantly inhibited by krill oil and other inhibitors at FDR < 0.05. Figure S4. Krill oil acted synergistically with COX2 and IKK2 inhibitors in increasing the expression of PPARG and FABP5 in human THP1 cells differentiated using PMA. Figure S5. The expression of IL17RA was significantly inhibited by krill oil in human THP1 cells in vitro. Figure S6. The number of bacteria engulfed by human macrophage phagocytosis was marginally increased by krill oil in vitro. Figure S7. A. A heat map showing the genes displaying a significant difference in abundance in the four experimental groups in the proximal colon tissue in pigs in response to Trichuris suis infection and dietary supplements. SC: uninfected pigs fed SO. SI: infected pigs fed SO. KC: uninfected pigs fed KO. KI: infected pigs fed KO. B. Modules-trait relationships in the signed consensus network. The correlation between pathophysiological traits, worm count, gut histamine levels, and gut fatty acid (FA_22:6) measurements, and the module eigengene value was calculated based on Pearson correlation. C. A scatterplot showing gene significance (y-axis) vs. module membership (x-axis) in the purple module. D. Transcription factors significantly enriched in the purple module in the signed consensus network. Figure S8. ANOSIM analysis of beta diversity in the porcine pro [file 40168_2020_843_MOESM1_ESM.docx]

**Mechanistic insights into the attenuation of intestinal inflammation and modulation of the gut microbiome by krill oil using in vitro and in vivo models**

Fang Liu, Allen D. Smith, Gloria Solano-Aguilar, Thomas T. Y. Wang, Quynhchi Pham,

Qingjuan Tang, Joseph F. Urban, Jr., Changhu Xue, Robert W. Li

**Supplementary information**

**Figure S1.** A PCA plot showing that krill oil (KO), COX2 inhibitor (celecoxib) and IKK2 inhibitor (TPCA1) induced distinct transcriptome patterns in human THP1 cells differentiated using phorbol 12-myristate 13-acetate (PMA).


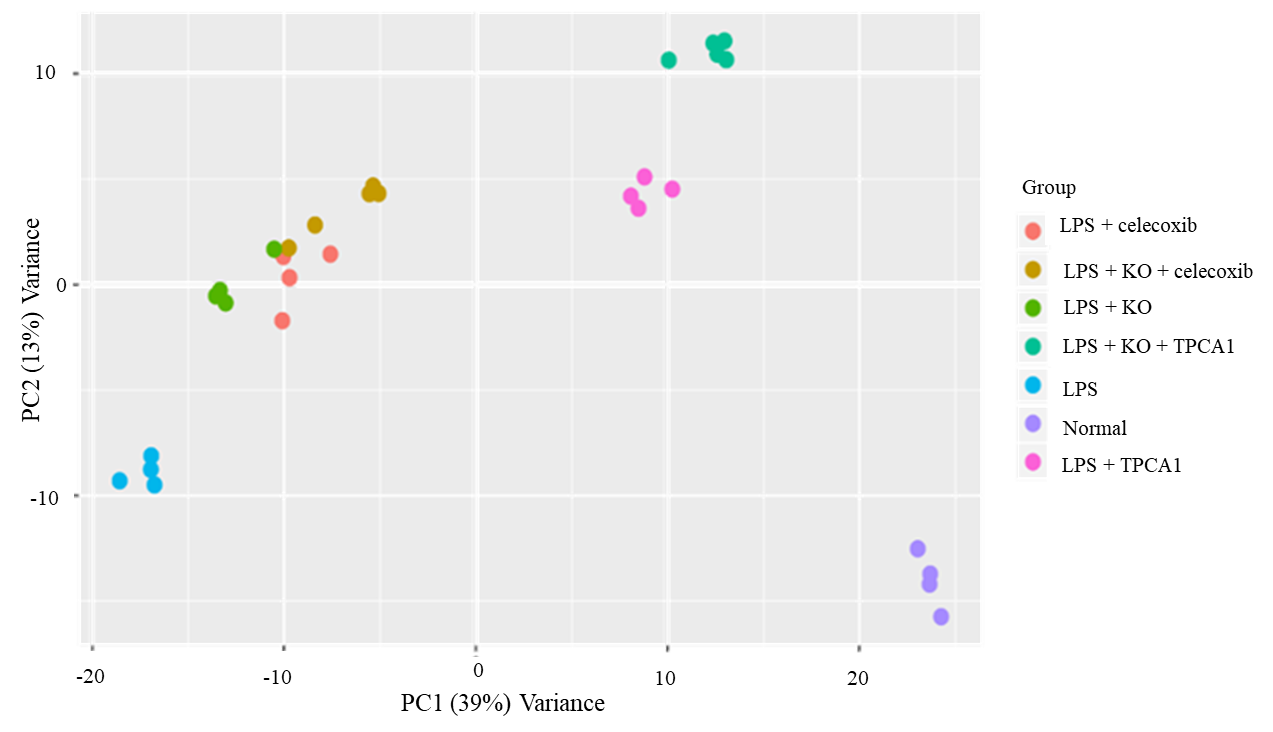


**Figure S2.** MA plots (log ratio vs mean average) for the visualization of differences in global transcriptome features of THP1 cells under different treatment conditions. The percentage denotes the number of up (red) or down (green) regulated genes induced by various compounds at a false discovery rate (FDR) < 0.05.


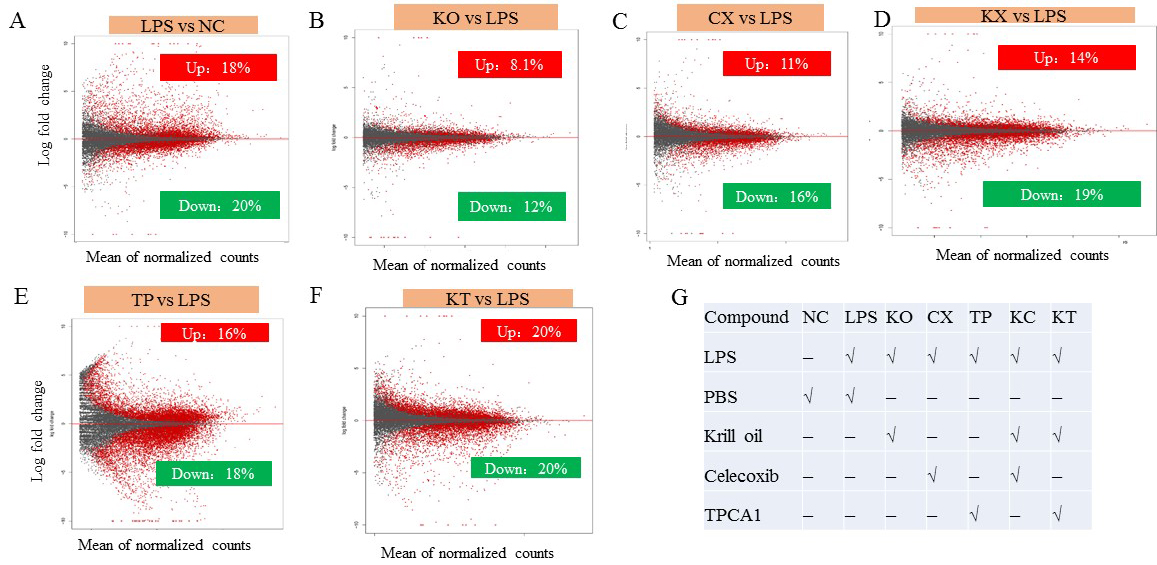


**Figure S3.** Heatmaps showing genes in Chemokine (A) and Nod-like receptor signaling (B) pathways significantly inhibited by krill oil and other inhibitors at FDR < 0.05.

**
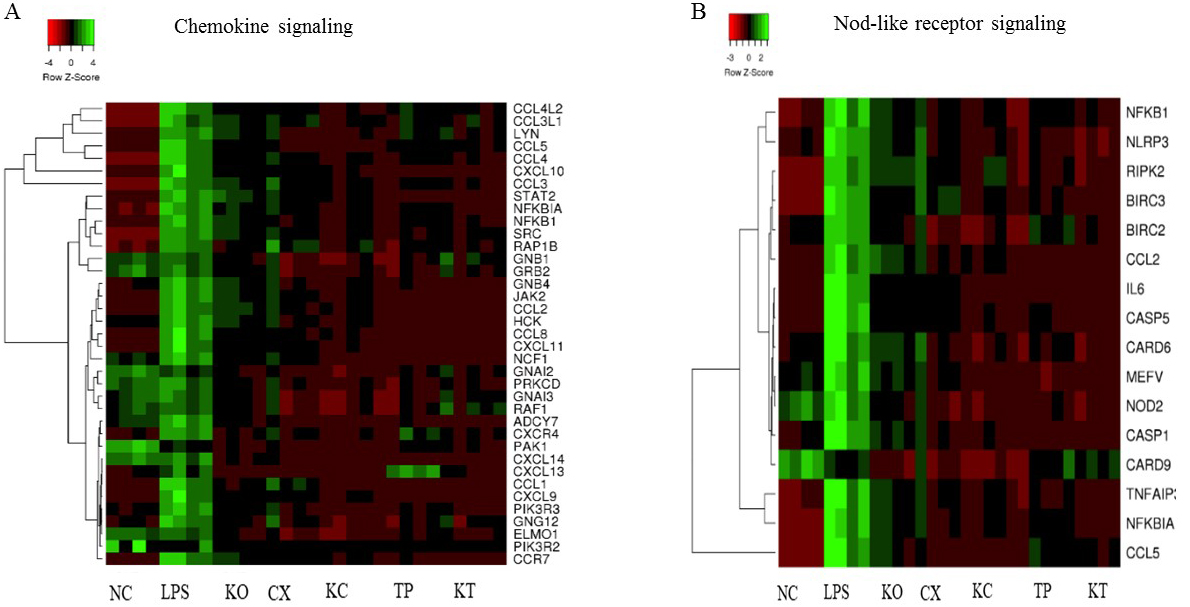
**

**Figure S4.** Krill oil acted synergistically with COX2 and IKK2 inhibitors in increasing the expression of PPARG and FABP5 in human THP1 cells differentiated using PMA.

**
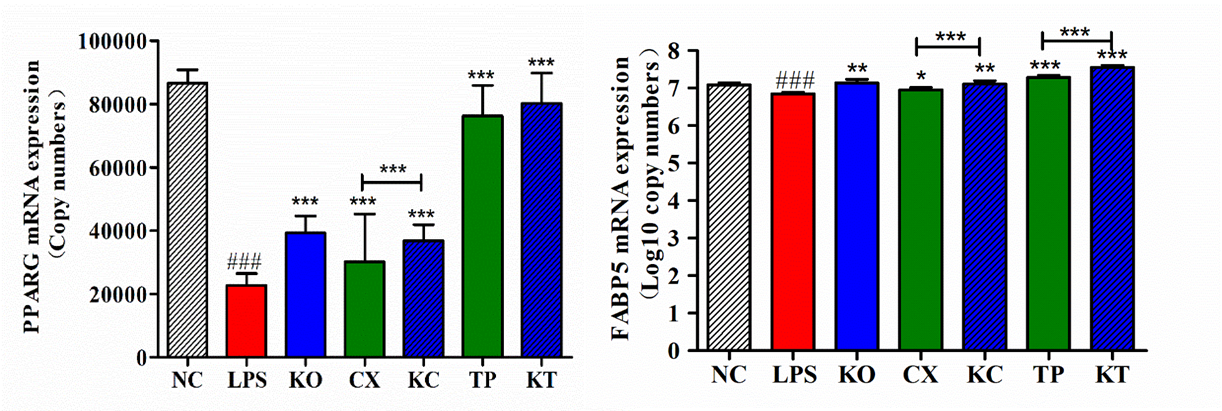
**

**Figure S5.** The expression of IL17RA was significantly inhibited by krill oil in human THP1 cells *in vitro*.

**

**

**Figure S6.** The number of bacteria engulfed by human macrophage phagocytosis was marginally increased by krill oil *in vitro*.

**

**

**Figure S7A.** A heat map showing the genes displaying a significant difference in abundance in the four experimental groups in the proximal colon tissue in pigs in response to *Trichuris suis* infection and dietary supplements. Red: downregulated; Yellow: upregulated. The intensity indicates the extent of fold changes. SC: uninfected animals supplemented with soybean oil (SO). SI: infected animals supplemented with SO. KC: uninfected animals supplemented with krill oil (KO). KI: infected animals supplemented with KO.

**
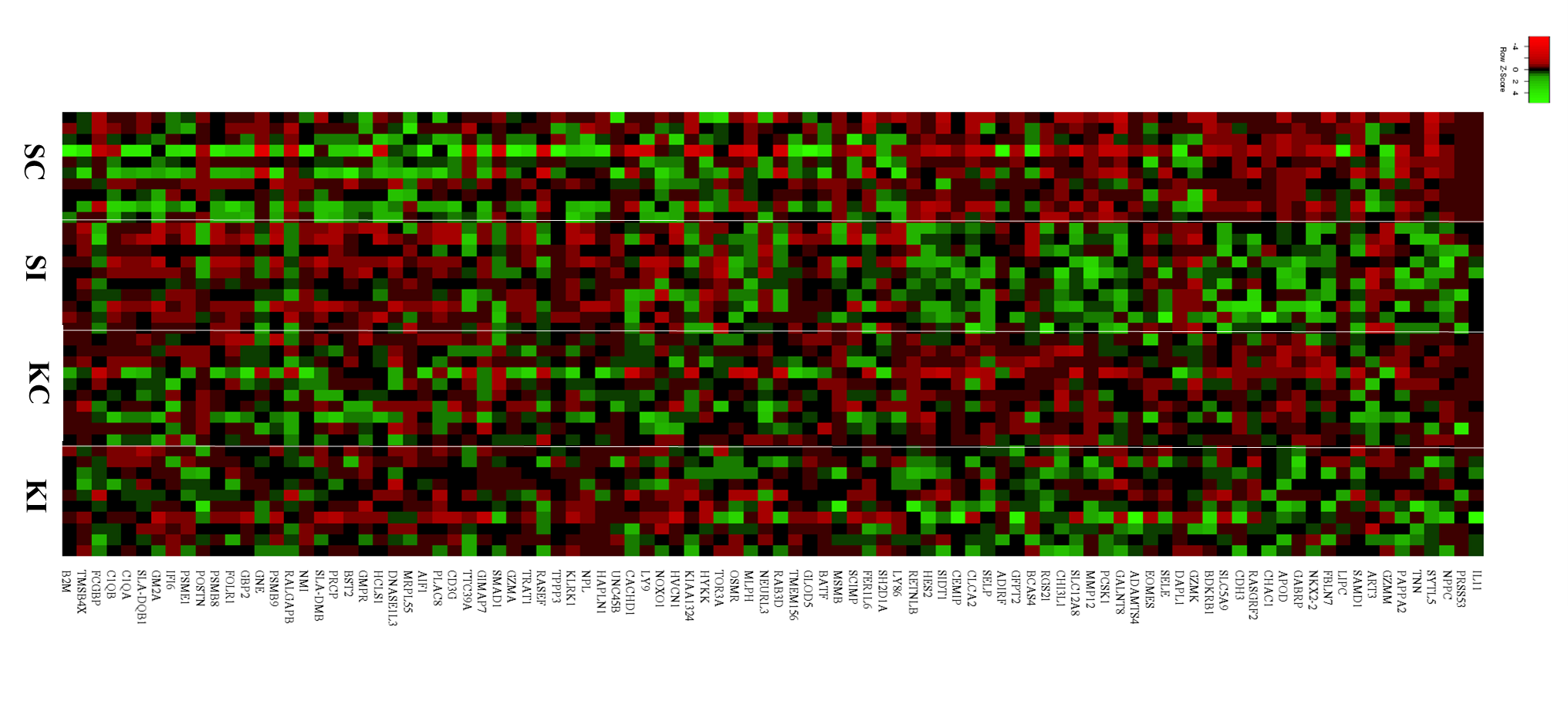
**

**Figure S7B.** Modules-trait relationships. The correlation between pathophysiological traits, worm count, gut histamine levels, and gut fatty acid (FA_22:6) measurements, and the module eigengene value was calculated based on Pearson correlation. Correlation coefficients along with *p* values (parenthesis underneath) were listed. Module color labels are shown on the left.

**Figure S7C.** A scatterplot showing gene significance (*y*-axis) vs. module membership (*x*-axis) in the module purple.

**Figure S7D. Transcription factors significantly enriched in the purple module in the signed consensus network. The genes on the left were hub genes in the purple module. The genes on the top were transcription factors enriched in the module.**

**
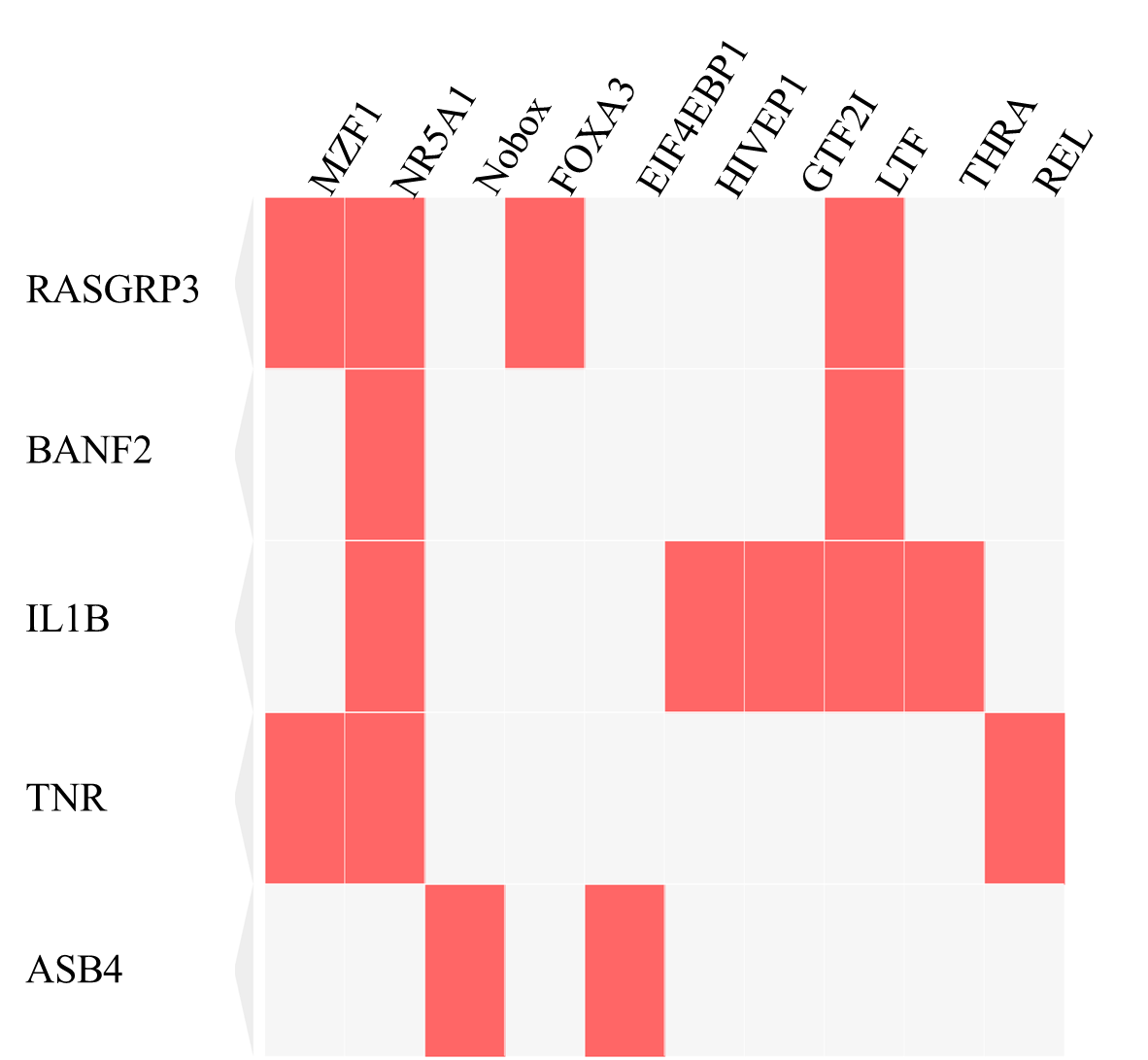
**

**Figure S8.** ANOSIM analysis of beta diversity in the porcine proximal colon microbiome. KO: krill oil. SO: soybean oil.

**
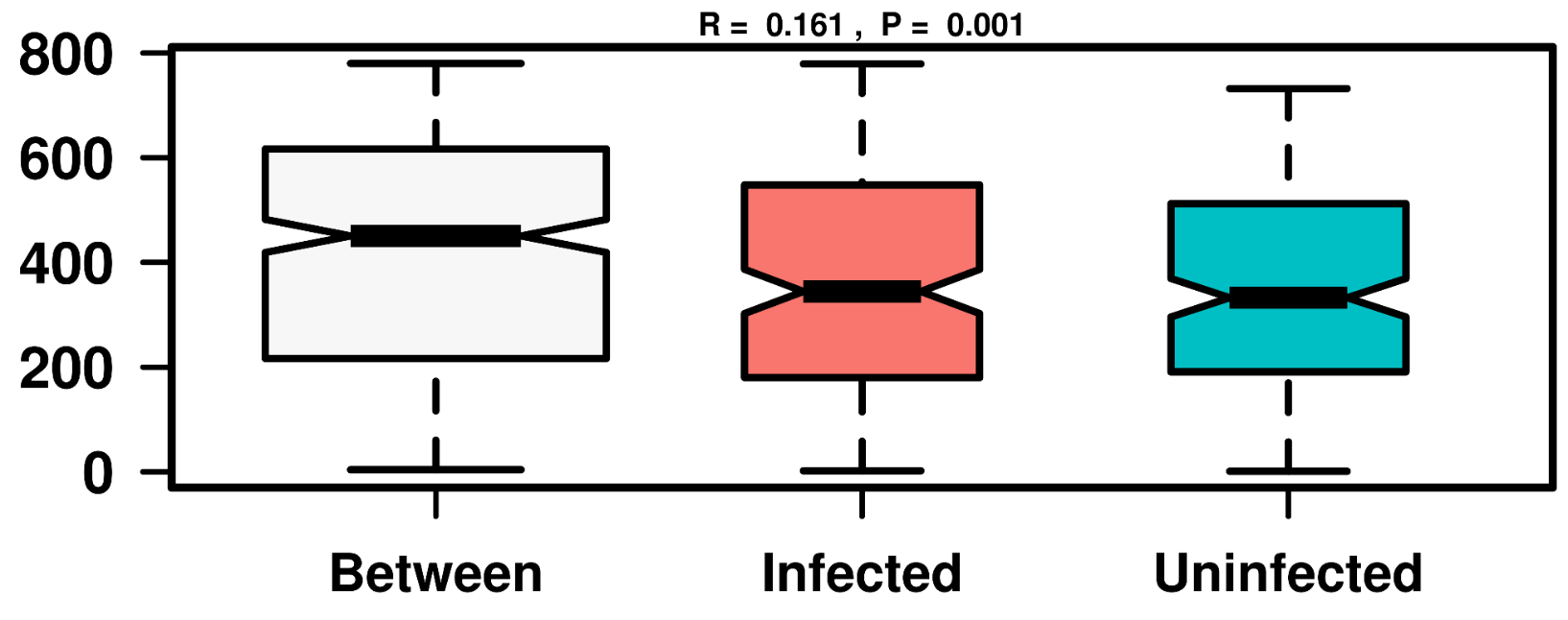
**

**
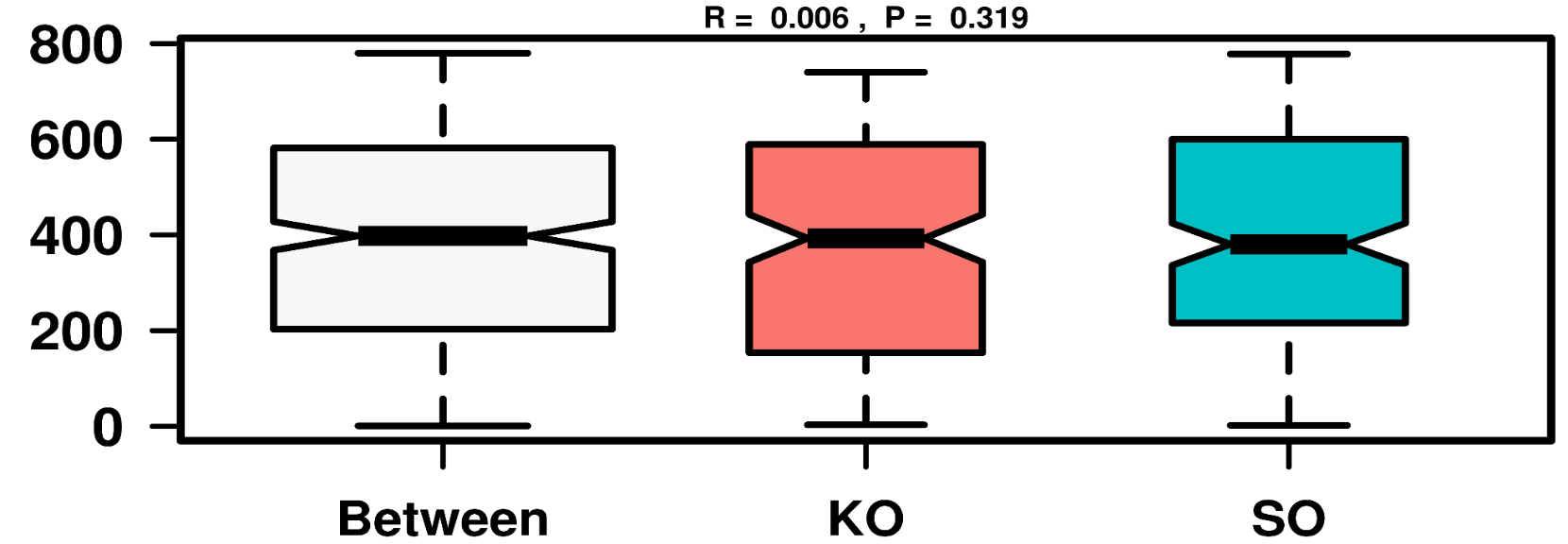
**

**Figure S9.** Global microbial interaction networks inferred using a Random Matrix Theory (RMT) based algorithm in the porcine proximal colon microbial community. SC: uninfected animals supplemented with soybean oil (SO). SI: infected animals supplemented with SO. KC: uninfected animals supplemented with krill oil (KO). KI: infected animals supplemented with KO. A dot represents an individual OTU. Colors represent different phyla.

**
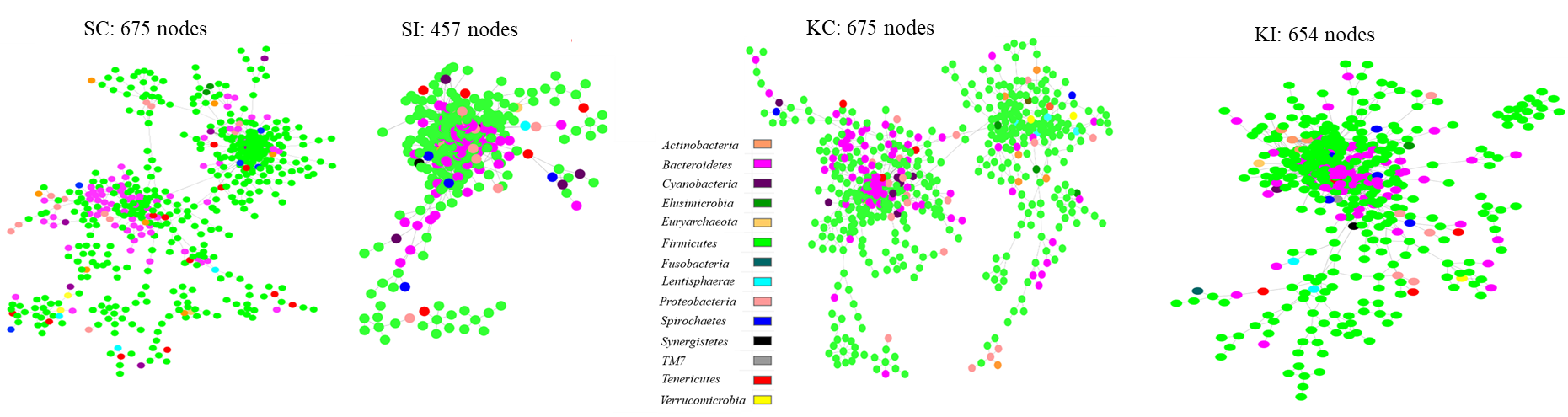
**

**Figure S10.** Microbial signatures or balances associated with acetate and eicosapentaenoic acid (EPA) concentrations in proximal colon contents in pigs. Y axis: Response variable: Acetate (A) and EPA (B).

**
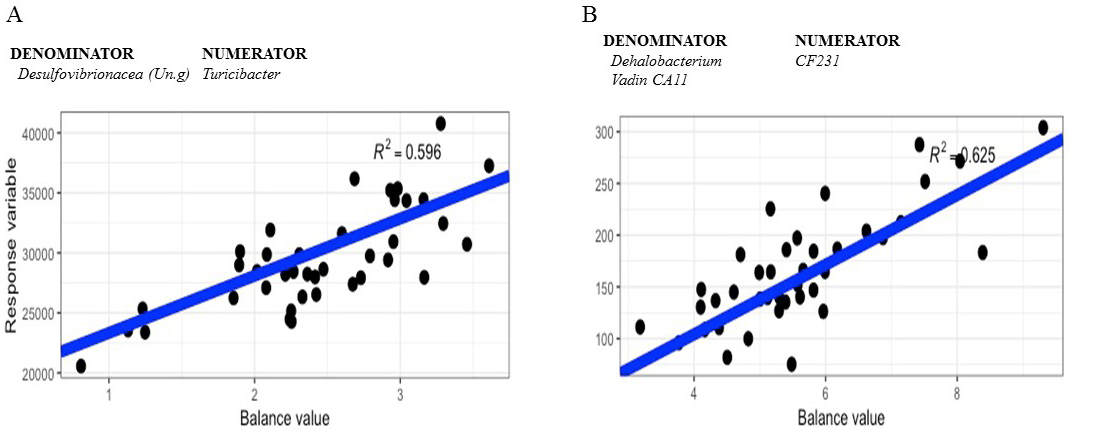
**

**Figure S11.** Select pathways significantly correlated with soybean (SO) and krill oil (KO) supplements.

**
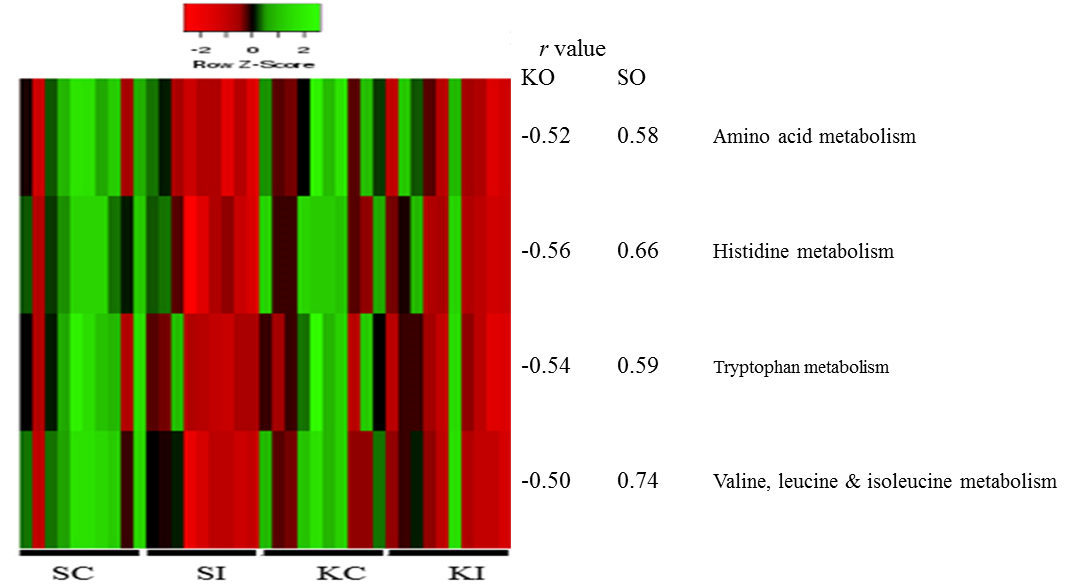
**

**Figure S12:** Taxa significantly correlated with gut luminal concentrations of histamine, 1-methyhistamine, and/or cir-urocanate in a *Citrobacter rodentium* induced murine colitis model. A: Genera. B: *Lactobacilus vaginalis*.

**
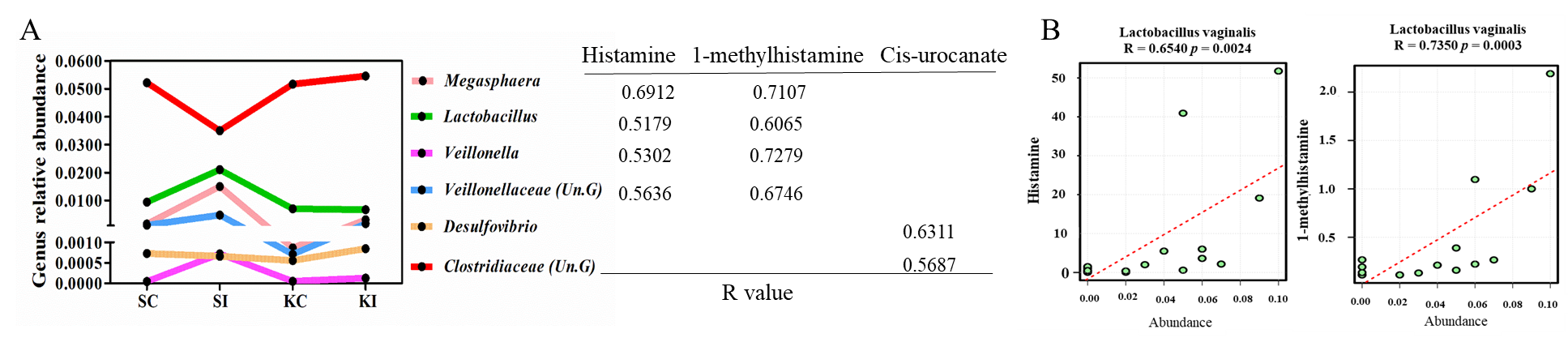
**

**Figure S13.** *Citrobacter rodentium* infection in mice had a significant impact on gut microbial diversity. A: Alpha diversity. B: Beta diversity (PCoA based on a distance matrix derived from Jaccard Index). NC: Uninfected healthy mice supplemented with PBS. CM: *Citrobacter rodentium* infected mice supplemented with PBS. KO: *Citrobacter rodentium* infected mice supplemented with krill oil. * *P* < 0.05; ** *P* < 0.01; *** *P* < 0.001.

**
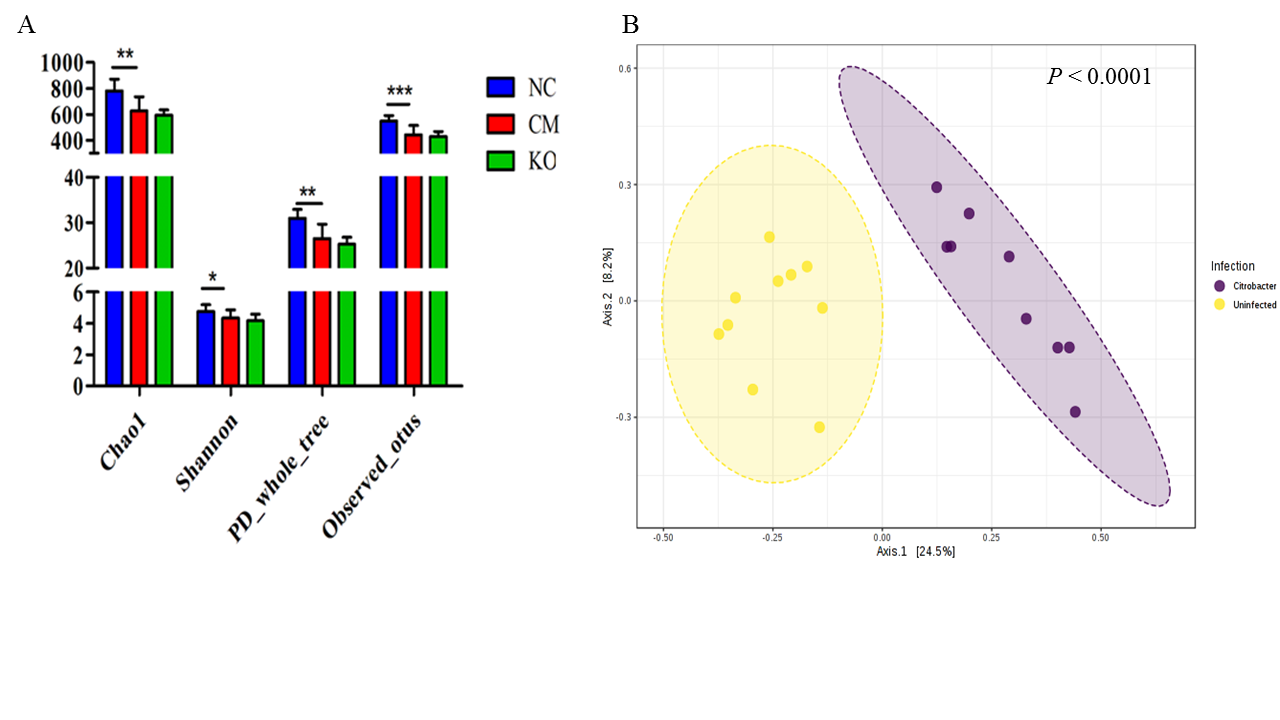
**

**Table S1.** Composition analysis of krill oil and soybean oil used in the study.

| **Krill oil** | **Results** | **Unit** |
| --- | --- | --- |
| Total phospholipids | 41.0 | g/100g |
| Total Omega-3 | 31.5 | g/100g |
| EPA | 23.3 | g/100g |
| DHA | 13.4 | g/100g |
| Total trans fat | 0.1 | g/100g |
| Astaxanthin (esterified) | 873.0 | mg/kg |
| Saponification | 174.0 | mg/KOH/g |
| Peroxide | < 0.2 | eEq/kg |
| Anisidine | 2.4 |  |
| Total oxidation value | 2.8 |  |
| Total aerobic bacterial count | 80.0 | CFU/g |
| Total coliform count | < 10 | CFU/g |
| Yeast & molds | < 10 | CFU/g |
| *E. coli* count | 0.0 | per 10g |
| *Staphylococcus aureus* | 0.0 | per 10g |
| *Listeria monocytogenes* | 0.0 | per 10g |
| *Pseudomonas aeruginosa* | 0.0 | per 10g |
| *Salmonella spp.* | 0.0 | per 10g |
| Moisture and volatile | 0.2 | % |
| Viscosity | 1173.0 | cP |
| Density | 0.98 | g/ml |

| **Soybean oil** | **Results** | **Unit** |
| --- | --- | --- |
| Free Fatty Acid | 0.04 | % |
| Peroxide Value | 0.17 | meq/kg |
| Fatty Acid Profile (Area %) |  |  |
| C16:0 Palmitic | 10.40 | % |
| C18:0 Stearic | 4.52 | % |
| C18:1w9 Oleic | 25.79 | % |
| C18:2w6 Linoleic | 52.28 | % |
| C18:3w3 Linolenic | 5.37 | % |

**Table S2.** Top 20 genera selected by Random Forest that distinguish the infection status in a porcine model.

| Genus | Mean Decrease Accuracy | Mean Decrease Gini | Fold  (Infected/Uninfected) |
| --- | --- | --- | --- |
| *Faecalibacterium* | 5.94 | 0.86 | 2.18 |
| *[Eubacterium]* | 5.70 | 0.62 | 2.07 |
| *Parabacteroides* | 5.07 | 0.71 | 0.40 |
| *Prevotella* | 5.02 | 0.69 | 1.36 |
| *Corynebacterium* | 4.79 | 0.48 | 0.17 |
| *Treponema* | 4.68 | 0.80 | 0.28 |
| *Fibrobacter* | 4.40 | 0.87 | 0.35 |
| *unclassified.WPS-2* | 4.36 | 0.41 | 0.19 |
| *unclassified.RFP12* | 4.11 | 0.51 | 0.19 |
| *YRC22* | 3.80 | 0.49 | 0.13 |
| *Anaerovibrio* | 3.58 | 0.22 | 0.62 |
| *Ruminococcus* | 3.09 | 0.38 | 0.51 |
| *rc4-4* | 2.98 | 0.31 | 0.42 |
| *unclassified.Dethiosulfovibrionaceae* | 2.97 | 0.33 | 0.51 |
| *Catenibacterium* | 2.81 | 0.30 | 2.69 |
| *Turicibacter* | 2.47 | 0.12 | 0.70 |
| *unclassified.Clostridiales* | 2.44 | 0.24 | 0.73 |
| *Peptococcus* | 2.40 | 0.24 | 0.75 |
| *unclassified.Tremblayales* | 2.33 | 0.23 | 0.13 |
| *Oscillospira* | 2.32 | 0.26 | 0.59 |

**Table S3.** Serum long chain polyunsaturated fatty acid (LCFA) in pigs. KO: krill oil. SO: soybean oil. HMDB: The Human Metabolome Database.

| **HMDB_ID** | **Serum LCFA** | KO mean | SO mean | KO/SO Fold | *P* value (Wilcoxon rank sum) |
| --- | --- | --- | --- | --- | --- |
|  |  |  |  |  |  |
|  |  |  |  |  |  |
| HMDB61714 | docosadienoate (22:2n6) | 1.1414 | 1.0297 | 1.1085 | 0.7197 |
| HMDB02183 | docosahexaenoate (DHA; 22:6n3) | 2.0442 | 0.792 | 2.5812 | 0.0015 |
| HMDB06528 | docosapentaenoate (DPA; 22:5n3) | 1.2387 | 0.9091 | 1.3626 | 0.0535 |
| HMDB01976 | docosapentaenoate  (n6 DPA; 22:5n6) | 0.8086 | 1.1782 | 0.6863 | 0.0279 |
| HMDB01999 | eicosapentaenoate (EPA; 20:5n3) | 1.8381 | 0.724 | 2.5387 | 0.0007 |

**Table S4.** The metabolites related to Histidine Metabolism was significantly affected by krill oil supplementation (KO) in pigs infected by *Trichuris suis*. SC: uninfected animals supplemented with soybean oil (SO). SI: infected animals supplemented with SO. KC: uninfected animals supplemented with KO. KI: infected animals supplemented with KO.

SI vs SC KI vs SI

| Metabolite | Fold | *P* value | Fold | *P* value |
| --- | --- | --- | --- | --- |
| 1-methyl-4-imidazoleacetate | 1.94 | 0.0426 | 0.49 | 0.0341 |
| 1-methyl-5-imidazoleacetate | 0.61 | 0.0004 | 1.34 | 0.0289 |
| 1-methylhistamine | 6.40 | 0.0173 | 0.20 | 0.0226 |
| 1-ribosyl-imidazoleacetate | 6.48 | 0.1646 | 0.15 | 0.1646 |
| 3-methylhistidine | 2.82 | 0.0033 | 1.19 | 0.3426 |
| 4-imidazoleacetate | 3.04 | 0.0025 | 0.68 | 0.0609 |
| cis-urocanate | 0.54 | 0.0019 | 2.18 | 0.0144 |
| formiminoglutamate | 0.98 | 0.4571 | 1.50 | 0.0149 |
| histamine | 59.13 | 0.0216 | 0.35 | 0.1257 |
| histidine | 2.05 | 0.0459 | 1.02 | 0.4810 |
| hydantoin-5-propionate | 1.30 | 0.1134 | 0.75 | 0.0793 |
| imidazole lactate | 4.57 | 0.1002 | 0.33 | 0.1356 |
| imidazole propionate | 4.91 | 0.0261 | 1.11 | 0.4151 |
| N-acetyl-1-methylhistidine | 2.81 | 0.0305 | 0.52 | 0.0897 |
| N-acetyl-3-methylhistidine | 0.75 | 0.0858 | 0.91 | 0.2348 |
| N-acetylhistamine | 24.72 | 0.0105 | 0.29 | 0.0434 |
| N-acetylhistidine | 1.92 | 0.0494 | 0.56 | 0.0591 |
| trans-urocanate | 1.08 | 0.3097 | 1.13 | 0.2061 |
